# Optogenetic modeling of human neuromuscular circuits in Duchenne muscular dystrophy with CRISPR and pharmacological corrections

**DOI:** 10.1126/sciadv.abi8787

**Published:** 2021-09-10

**Authors:** Amaia Paredes-Redondo, Peter Harley, Eleni Maniati, David Ryan, Sandra Louzada, Jinhong Meng, Anna Kowala, Beiyuan Fu, Fengtang Yang, Pentao Liu, Silvia Marino, Olivier Pourquié, Francesco Muntoni, Jun Wang, Ivo Lieberam, Yung-Yao Lin

**Affiliations:** 1 Centre for Genomics and Child Health, Blizard Institute, Barts and the London School of Medicine and Dentistry, Queen Mary University of London, 4 Newark Street, London E1 2AT, UK.; 2 Stem Cell Laboratory, National Bowel Research Centre, Blizard Institute, Barts and the London School of Medicine and Dentistry, Queen Mary University of London, 2 Newark Street, London E1 2AT, UK.; 3 Centre for Predictive in vitro Model, Queen Mary University of London, Mile End Road, London E1 4NS, UK.; 4 Centre for Stem Cells and Regenerative Medicine, MRC Centre for Neurodevelopmental Disorders, and Centre for Developmental Neurobiology, King’s College London, London, UK.; 5 Centre for Cancer Genomics and Computational Biology, Barts Cancer Institute, Queen Mary University of London, London, UK.; 6 Wellcome Sanger Institute, Wellcome Genome Campus, Hinxton, Cambridge CB10 1SA, UK.; 7 UCL Great Ormond Street Institute of Child Health, 30 Guilford Street, London WC1N 1EH, UK.; 8 School of Biomedical Sciences, Stem Cell and Regenerative Medicine Consortium, Li Ka Shing Faculty of Medicine, The University of Hong Kong, Hong Kong, China.; 9 Department of Genetics and Department of Pathology, Brigham and Women’s Hospital, Harvard Medical School, 60 Fenwood Road, Boston, MA, USA.; 10 NIHR Biomedical Research Centre, Great Ormond Street Hospital, Great Ormond Street, London, UK.

## Abstract

Duchenne muscular dystrophy (DMD) is caused by *dystrophin* gene mutations leading to skeletal muscle weakness and wasting. Dystrophin is enriched at the neuromuscular junction (NMJ), but how NMJ abnormalities contribute to DMD pathogenesis remains unclear. Here, we combine transcriptome analysis and modeling of DMD patient-derived neuromuscular circuits with CRISPR-corrected isogenic controls in compartmentalized microdevices. We show that NMJ volumes and optogenetic motor neuron–stimulated myofiber contraction are compromised in DMD neuromuscular circuits, which can be rescued by pharmacological inhibition of TGFβ signaling, an observation validated in a 96-well human neuromuscular circuit coculture assay. These beneficial effects are associated with normalization of dysregulated gene expression in DMD myogenic transcriptomes affecting NMJ assembly (e.g., *MUSK*) and axon guidance (e.g., *SLIT2* and *SLIT3*). Our study provides a new human microphysiological model for investigating NMJ defects in DMD and assessing candidate drugs and suggests that enhancing neuromuscular connectivity may be an effective therapeutic strategy.

## Teaser

SB-431542 restores neuromuscular synapses and motor function in a human microphysiological model of muscular dystrophy.

## INTRODUCTION

Duchenne muscular dystrophy (DMD), caused by mutations in the gene encoding dystrophin (*DMD*) on the X chromosome, is a fatal and the most common inherited neuromuscular disorder in childhood, affecting 1 in 3500 to 5000 live male births (*1*). The dystrophin-glycoprotein complex (DGC) maintains the integrity of skeletal muscle by linking the intracellular cytoskeleton to the extracellular matrix and participates in cellular signaling processes (*2*). Patients with DMD suffer from progressive skeletal muscle weakness and wasting that lead to eventual loss of ambulation with reduced life expectancy (*3*). Current standard of care for DMD is based on the palliative effect of corticosteroids, which increase muscle mass and strength while reducing inflammation and necrosis. Nevertheless, prolonged corticosteroid treatment is associated with side effects, including weight gain, osteoporosis, and cataracts (*4*). To date, only two types of curative medicines have received approval for treating DMD. Ataluren is a compound that induces ribosomal readthrough of premature termination codons to restore dystrophin protein expression, suitable for approximately 13% of patients with DMD, and approved by European Medicines Agency (*5*). Eteplirsen, golodirsen, viltolarsen, and casimersen are antisense oligonucleotides that mediate pre-mRNA exon skipping to restore the open reading frame in patients with DMD with eligible mutations (*6*, *7*), cumulatively allowing targeting approximately 30% of patients with DMD. Despite these existing drugs, there is still no cure or effective treatment for DMD.

The formation of neuromuscular circuits is critical for generating voluntary movement, in which skeletal muscle contraction is induced by motor neurons (MNs) through neurotransmitter release at neuromuscular junctions (NMJs), where motor axons form synaptic contacts with myofibers. The DGC is enriched at the plasma membrane of myofibers and NMJs (*8*). While research in DMD primarily focused on muscle wasting, studies have shown that both patients with DMD and dystrophin-deficient *mdx* mice have structural alterations at the NMJs and aberrant electrophysiological changes (*9*–*12*), suggesting that NMJ abnormalities may contribute to pathophysiology of DMD. Although dystrophin-deficient mice have been extensively used as an animal model to investigate mechanisms underlying DMD, the genetic and physiological differences between rodents and humans highlight the need to improve translatability of preclinical studies using the *mdx* mice for evaluating potential therapeutics. For example, in contrast to patients with DMD, the *mdx* mice have relatively mild muscle pathology, possibly due to compensatory or species-specific mechanisms in mice (*13*). Furthermore, human and mouse NMJs differ substantially in their cellular anatomy and synaptic proteomes (*14*). To reduce the use of animals and facilitate the selection of the most promising therapies for DMD clinical trials, it is important to develop human-specific and physiologically relevant models for preclinical studies of neuromuscular circuits in health and disease, as well as for assessing the efficacy of therapeutic strategies.

**Fig. 1.**
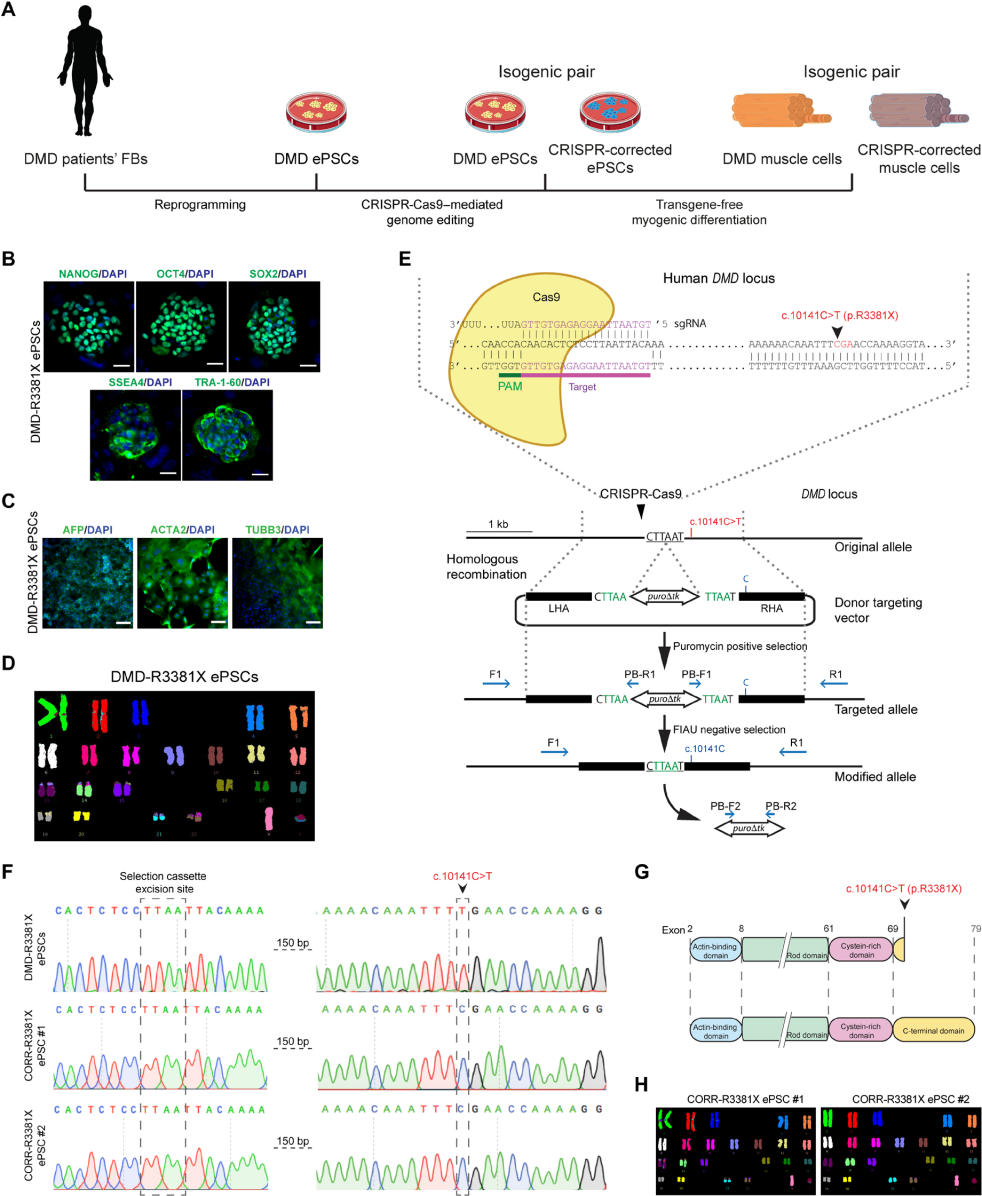
Generation and characterization of an isogenic pair of DMD patient-derived and CRISPR-corrected PSC lines. (**A**) Schematic of the generation of ePSCs from DMD patient-derived fibroblasts (FBs) and CRISPR-Cas9–mediated precise gene correction to generate isogenic control ePSCs, followed by myogenic differentiation to obtain muscle cells. (**B**) Representative immunocytochemistry images of pluripotency markers—NANOG, OCT4, SOX2, SSEA4, and TRA-1-60—in DMD-R3381X ePSCs. Scale bars, 100 μm. DAPI, 4′,6-diamidino-2-phenylindole. (**C**) In vitro differentiation of DMD-R3381X ePSCs to three embryonic germ layers, confirmed by positive immunocytochemistry of endoderm (α-fetoprotein, AFP), mesoderm (α-smooth muscle actin, ACTA) and ectoderm (β3-tubulin, TUBB3) markers. Scale bars, 100 μm. (**D**) Normal karyotype of the DMD-R3381X ePSCs. (**E**) Schematic diagram of gene correction in DMD-R3381X ePSCs. Cas9 protein and the specific single guide RNA (sgRNA) target the *DMD* locus 150 base pairs (bp) upstream the mutation. The donor targeting vector carries a *piggyBac* (*PGK-puro*∆*tk*) selection cassette flanked by two homology arms [left homology arm (LHA); right homology arm (RHA)]. Blue arrows indicate forward (F)/reverse (R) primers for genotyping and sequencing. PB, piggyBac; FIAU, Fialuridine. (**F**) Sequence analysis confirmed precise correction of the *DMD* c.10141C>T mutation in two independent clones, CORR-R3381X ePSC #1 and #2, without modification at the selection cassette excision site. (**G**) Schematic of the truncated dystrophin in DMD-R3381X cells and restored full-length dystrophin in CORR-R3381X cells. (**H**) Normal karyotype of the CORR-R3381X ePSC clones.

The advent of human pluripotent stem cells (PSCs) and transgene-free myogenic differentiation has opened new avenues of research into muscular dystrophies and drug discovery (*15*, *16*). For instance, patient-specific PSC-derived skeletal muscle has been shown to recapitulate some pathological features of DMD in monotypic two-dimensional (2D) cultures, including reduced myoblast fusion competence, abnormal expression of inflammation-related genes, and up-regulation of transforming growth factor–β (TGFβ)/bone morphogenetic protein signaling (*17*). Notably, these cellular phenotypes are variable from patient to patient, probably because of differences in individual genetic backgrounds. This highlights the importance of generating appropriate isogenic controls using recently developed genome editing tools (Fig. 1A) (*15*).

In contrast to conventional monotypic 2D cultures, recent advances in 3D multilineage cultures can better mimic the complex human tissue architecture and physiology in vivo (*15*). In particular, compartmentalized microdevices and organ-on-a-chip technologies have recently been exploited to recapitulate physiological conditions and model nerve-muscle connectivity in the context of MN disease, amyotrophic lateral sclerosis (ALS) (*18*, *19*). Furthermore, optogenetic stimulation through the light-gated ion channel, channelrhodopsin-2 (*20*), can specifically elicit synaptic activity of PSC-derived MNs, which, in turn, induces contraction of PSC-derived myofibers. The generation of simplified motor units in vitro enables preclinical testing of drug candidates that can ameliorate pathological features of ALS (*18*, *19*). By contrast, it remains poorly understood with regard to neuromuscular circuit formation in DMD and its impact on muscle contractility.

Here, we develop a patient-specific in vitro model of DMD with isogenic controls by integrating human PSC–derived myofibers and MNs, CRISPR-mediated genome editing, optogenetics, and microfabrication technologies. Transcriptome analysis of DMD myogenic cultures compared to CRISPR-corrected isogenic controls expressing full-length dystrophin reveals affected gene sets, including NMJ assembly and axon guidance. We report optogenetic modeling of neuromuscular circuits in DMD with isogenic controls in compartmentalized microdevices. We show that NMJ volumes and light-stimulated myofiber contraction are compromised in DMD neuromuscular circuits, which can be rescued by pharmacological inhibition of TGFβ signaling with independent validation in a 96-well human neuromuscular circuit coculture assay. In particular, many of the abnormally expressed genes affecting NMJ assembly (e.g., *MUSK*) and axon guidance (e.g., *SLIT2* and *SLIT3*) respond to inhibition of TGFβ signaling in DMD myogenic cultures. Our findings suggest that dysregulated gene expression of NMJ assembly and axon guidance in the DMD myogenic transcriptome may contribute to defective muscle-nerve connectivity in DMD, leading to skeletal muscle weakness. Together, optogenetic modeling of human neuromuscular circuits in vitro provides a microphysiological platform that can be further exploited for investigating mechanisms underlying DMD pathophysiology and assessing potential therapeutic strategies.

## RESULTS

### Generation and characterization of an isogenic pair of DMD patient-derived and CRISPR-corrected PSC lines

We obtained dermal fibroblasts from a DMD patient with clinical diagnosis summarized in table S1. Genetic analysis revealed the *DMD* c.10141C>T (p.R3381X) nonsense mutation in exon 70 (fig. S1A), affecting all tissue-specific dystrophin isoforms (Dp427, Dp260, Dp140, Dp116, and Dp71). To generate DMD PSCs, we reprogrammed the DMD patient’s fibroblasts to a pluripotent state using a six-factor reprogramming technology and a recently developed expanded potential stem cell medium (EPSCM) (*21*, *22*). DMD patient-derived PSCs stably maintained in EPSCM are referred to as DMD-R3381X EPSCM-PSC (hereinafter ePSCs). Initial characterization of two independent DMD-R3381X ePSCs clonal lines confirmed expression of pluripotency markers by quantitative polymerase chain reaction (qPCR; fig. S1B). Immunocytochemistry also confirmed expression of pluripotency markers, including Nanog homeobox (NANOG), POU class 5 homeobox 1 (OCT4), SRY-box transcription factor 2 (SOX2), Stage-specific embryonic antigen 4 (SSEA4), and T cell receptor alpha locus 1-60 (TRA-1-60). (Fig. 1B). The DMD-R3381X ePSC line underwent in vitro differentiation to form embryoid bodies (EBs). Positive immunocytochemistry for markers of each of the three embryonic germ layers confirmed DMD-R3381X clone pluripotent status (Fig. 1C), and analysis of the karyotype revealed no chromosomal abnormalities (Fig. 1D). To generate isogenic control cells, we precisely corrected the *DMD* c.10141C>T mutation in the DMD-R3381X clone using CRISPR-Cas9–mediated genome editing technology. The strategy was based on the homologous directed repair pathway activated in the cells after a double-strand break in the DNA (Fig. 1E), using a double selection approach based on a *piggyBac* transposon-based selection cassette (*PGK-puro*∆*tk*) to facilitate the screening for targeted and edited events as previously described (*23*). Sequencing of corrected clones (hereinafter CORR-R3381X ePSCs) confirmed precise correction of the *DMD* mutation without any alteration in the selection cassette excision site (Fig. 1, F and G). Microsatellite analysis demonstrated the common origin of DMD-R3381X and the two CRISPR-corrected clones (fig. S1C). Karyotype analysis showed no chromosomal abnormalities after genome editing (Fig. 1H). Sequencing of the top five predicted potential off-target sites suggested that no mutations were introduced during genome editing (fig. S1D). Last, immunocytochemistry showed maintenance of pluripotency by expression of NANOG, OCT4, SOX2, SSEA4, and TRA-1-60 in the CRISPR-corrected ePSCs (fig. S1E).

### Restoration of full-length dystrophin in CRISPR-corrected myogenic cultures is associated with higher differentiation competence than DMD

**Fig. 2.**
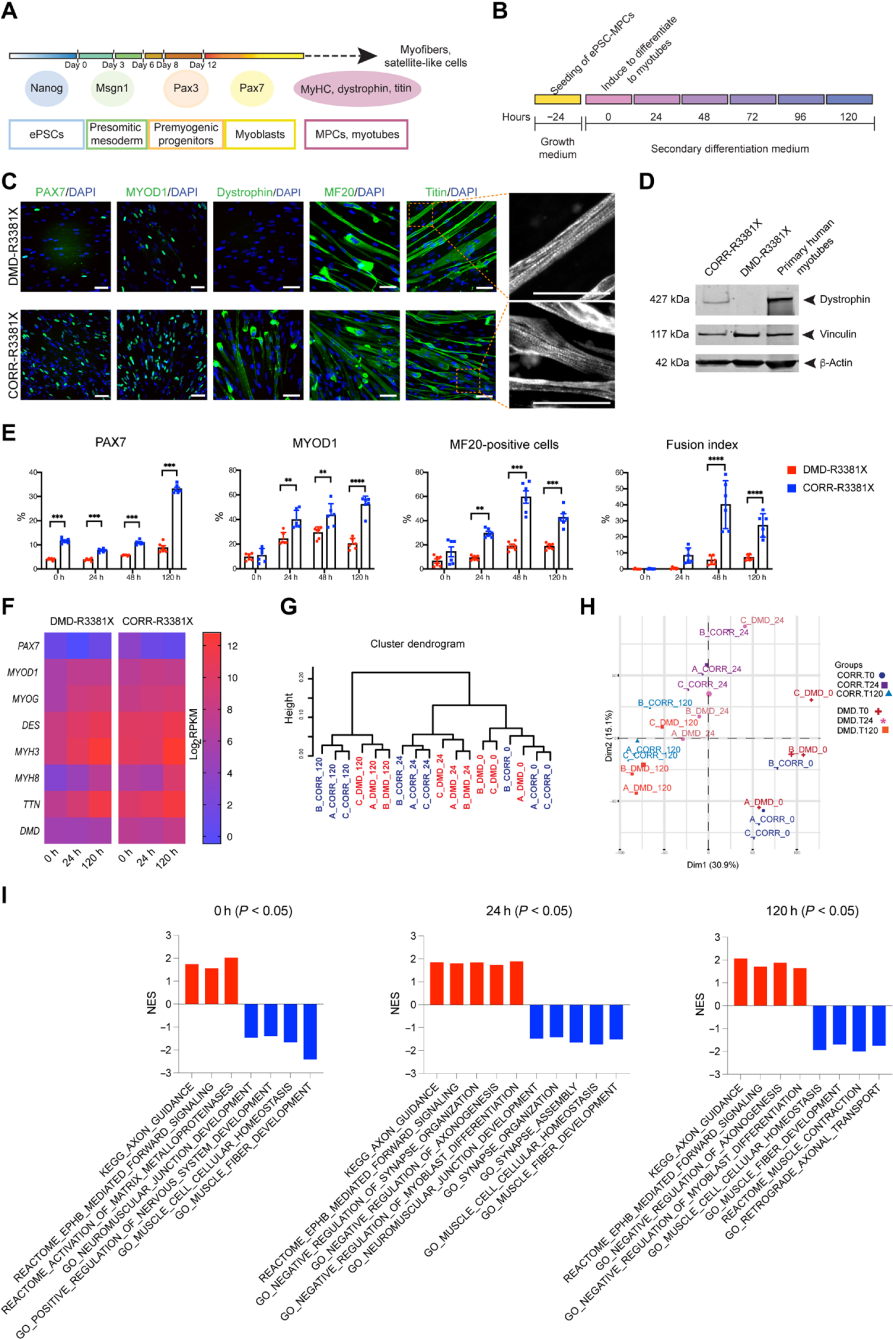
Characterization of DMD and CRISPR-corrected PSC-derived MPCs and myotubes. (**A**) Timeline of the myogenic specification from human ePSCs to MPCs and myotubes with markers for each specific stage. (**B**) Secondary differentiation of MPCs into myotubes. Experiments are analyzed at 0 hours (MPCs in skeletal muscle cell growth medium) and at 24, 48, and 120 hours after switching to secondary differentiation medium. (**C**) Representative immunocytochemistry images of PAX7, MYOD1, dystrophin, MYH (MF20), and titin in DMD-R3381X and CORR-R3381X myogenic cultures at 120 hours of secondary differentiation. Enlarged images of titin demonstrate sarcomere striation. Scale bars, 100 μm. (**D**) Immunoblotting confirms that full-length dystrophin (427 kDa) is restored in CORR-R3381X myogenic cultures. (**E**) Quantification of PAX7-positive, MYOD1-positive, and MF20-positive cells and fusion index during secondary differentiation at 0, 24, 48, and 120 hours in DMD-R3381X and CORR-R3381X cultures. Values are means ± SD. *n* = 6. Two-way analysis of variance (ANOVA) and Sidak’s multiple comparisons test, **P < 0.01, ***P < 0.001, and ****P < 0.0001. (**F**) Heatmap of normalized gene expression levels as log_2_ transformed RPKM (reads per kilobase per million mapped reads) of myogenic markers in DMD-R3381X and CORR-R3381X cells at 0, 24, and 120 hours of secondary differentiation. (**G**) Unsupervised cluster dendrogram of DMD-R3381X and CORR-R3381X transcriptomes at 0, 24, and 120 hours of secondary differentiation. (**H**) A principal components analysis graph of DMD-R3381X and CORR-R3381X transcriptomes at 0, 24, and 120 hours of secondary differentiation. (**I**) Selected gene sets with statistically significant normalized enrichment score (NES) in DMD-R3381X (red bars) or CORR-R3381X (blue bars) myogenic cultures at 0, 24, and 120 hours of secondary differentiation.

To model DMD in vitro, we differentiated the isogenic pair of DMD and CRISPR-corrected ePSCs to myogenic progenitor cells (MPCs) and myotubes using a transgene-free protocol as described (*24*). This protocol has a primary differentiation phase, in which the ePSCs differentiate to MPCs, followed by subculture in skeletal muscle growth medium and cryopreservation (Fig. 2A). In the secondary differentiation phase, the ePSC-derived MPCs are induced to form myotubes with self-renewed MPCs in skeletal muscle differentiation medium (Fig. 2B). Immunocytochemistry results revealed that both DMD-R3381X and CORR-R3381X ePSC-derived MPCs expressed the myogenic transcription factors paired box 7 (PAX7) and myogenic differentiation 1 (MYOD1) and myotubes derived from the isogenic pair of MPCs expressed sarcomeric proteins, such as myosin heavy chain (MYH) and titin (Fig. 2C). Immunocytochemistry of CORR-R3381X myotubes showed restored expression of dystrophin protein after precise gene correction (Fig. 2C). Immunoblotting confirmed that the molecular mass of dystrophin protein in CORR-R3381X myogenic culture is 427 kDa, consistent with the full-length dystrophin muscle isoform Dp427 (Fig. 2D). Although conventional immunoblotting was not sensitive enough to detect the dystrophin isoform Dp71 in skeletal muscle (*25*), the presence of Dp427 in CORR-R3381X implies that Dp71 protein expression should also be restored.

Quantification of PAX7-positive nuclei during secondary differentiation showed significant differences between DMD-R3381X and CORR-R3381X in all time points 0, 24, 48, and 120 hours (Fig. 2E and fig. S2A). The percentage of PAX7-positive nuclei in DMD-R3381X cultures remained constantly below 10% with no significant change over time, whereas PAX7-positive nuclei in CORR-R3381X culture increased about threefold at 120 hours (Fig. 2E). Quantification of MYOD1-positive nuclei at 0 hours showed no significant difference between DMD-R3381X and CORR-R3381X (Fig. 2E and fig S2B). During secondary differentiation, both lines showed an upward trend in the percentage of MYOD1-positive nuclei; however, DMD-R3381X cultures had a significantly lower percentage of MYOD1-positive nuclei compared to CORR-R3381X cultures at the 24, 48, and 120 hours (Fig. 2E). These results may be, in part, explained by a recent finding that a Dp71 variant is expressed in human satellite cells and enhances myoblast proliferation (*26*). Next, we investigated the differentiation and fusion capacity of DMD-R3381X and CORR-R3381X MPCs by quantifying the percentage of MYH-positive cells and fusion index (Fig. 2E and fig. S2C). While there was no significant difference at 0 or 24 hours of secondary differentiation, CORR-R3381X cultures efficiently differentiated to multinucleated myotubes with significantly higher MYH-positive cells and fusion index compared to DMD-R3381X cultures at 48 and 120 hours (Fig. 2E). Together, these data suggest a reduced differentiation and fusion competence of dystrophin-deficient MPCs.

### Analysis of myogenic transcriptomes identifies gene sets affected in DMD

Next, we sought to investigate whether the reduced differentiation and fusion of DMD-R3381X cells were due to aberrant myogenic gene expression. We performed transcriptome sequencing and analysis of DMD-R3381X and CORR-R3381X myogenic cultures during secondary differentiation (0, 24, and 120 hours). By examining transcript levels in DMD-R3381X compared to CORR-R3381X myogenic cultures, we observed lower expression of *PAX7* in DMD-R3381X at time point 0 hours (*P* = 0.017, log_2_FC = −4.14). This difference was, however, not retained at time points 24 and 120 hours (fig. S2D). Similarly, there was no significant difference in *MYOD1* transcript levels between the two genotypes during secondary differentiation (fig. S2E). Contrary to lower percentages of PAX7- and MYOD1-positive nuclei in DMD-R3381X cultures (Fig. 2E), these results suggest that dystrophin deficiency may have an effect on translation of *PAX7* and *MYOD1* or as yet unidentified mechanisms, although this will require further investigation. Further analysis revealed similar expression profiles of a number of muscle-specific genes between DMD-R3381X and CORR-R3381X myogenic cultures, including the transcription factor *MYOG* and sarcomeric components such as desmin (*DES*), MYH isoforms 3 and 8 (*MYH3* and *MYH8*), and titin (*TTN*) (Fig. 2F). Notably, DMD-R3381X cultures had significantly decreased *DMD* transcript levels compared to CORR-R3381X at each stage (Fig. 2F and fig. S2F), which may be, in part, explained by a recent discovery involving epigenetic regulation (*27*).

Unsupervised sample clustering by hierarchical cluster analysis (Fig. 2G) confirmed clustering of biological replicates (A, B, and C), segregation of the samples in relation to their stages of differentiation (0, 24, and 120 hours), and segregation of the samples according to genotype (DMD-R3381X and CORR-R3381X). Genotype segregation was evident at 24- and 120-hour time points. At the beginning of secondary differentiation (time point, 0 hours), we observed that DMD-R3381X samples interspersed with CORR-R3381X. This indicates that the transcriptome differences between these two genotypes become more pronounced as the differentiation program advances. In line with this, principal components analysis indicated approximately 46% of variance in the samples (Dim1 = 30.9% and Dim2 = 15.1%) could be explained by the stage of secondary differentiation and, to a lesser extent, by genotype (Fig. 2H).

To further elucidate mechanisms underlying DMD, we identified differentially expressed genes between DMD-R3381X and CORR-R3381X and carried out gene set enrichment analysis (GSEA). Gene sets with positive normalized enrichment score (NES) reflected overall higher transcript levels in DMD-R3381X transcriptomes compared to CORR-R3381X, whereas gene sets with negative NES indicated overall higher transcript levels in CORR-R3381X transcriptomes than DMD-R3381X. We considered NES with nominal *P* < 0.05 as significantly enriched gene sets, which reflect many characteristic DMD pathophysiology, e.g., inflammation, Ca^2+^ homeostasis, and mitochondrial metabolism (data files S1 and S2). Consistent with the immunocytochemistry results of myogenic markers, gene sets associated with CORR-R3381X transcriptomes during secondary differentiation included Gene Ontology (GO) muscle fiber development and GO muscle cell cellular homeostasis (Fig. 2I). Furthermore, at 120 hours of secondary differentiation, Reactome Muscle contraction was associated with CORR-R3381X, whereas GO negative regulation of myoblast differentiation was correlated with DMD-R3381X (Fig. 2I). We noticed that gene sets modulating NMJ assembly were correlated with CORR-R3381X, such as GO neuromuscular junction development, GO synapse organization, and GO synapse assembly, while GO negative regulation of synapse organization was associated with DMD-R3381X at 24 hours of secondary differentiation (Fig. 2I). In addition, gene sets regulating nerve function were correlated with DMD-R3381X at all three time points, including Kyoto Encyclopedia of Genes and Genomes (KEGG) Axon guidance and Reactome EPHB-mediated forward signaling (Fig. 2I). In particular, GO negative regulation of axonogenesis was correlated with DMD-R3381X at 24 and 120 hours of secondary differentiation. In contrast, GO retrograde axonal transport was correlated with CORR-R3381X at 120 hours (Fig. 2I). Together, our findings suggest that abnormal gene expression profiles in DMD myogenic transcriptomes may affect not only skeletal muscle differentiation and homeostasis but also synapse organization and axonogenesis.

### Assembly of DMD and CRISPR-corrected neuromuscular circuits in compartmentalized microdevices

**Fig. 3.**
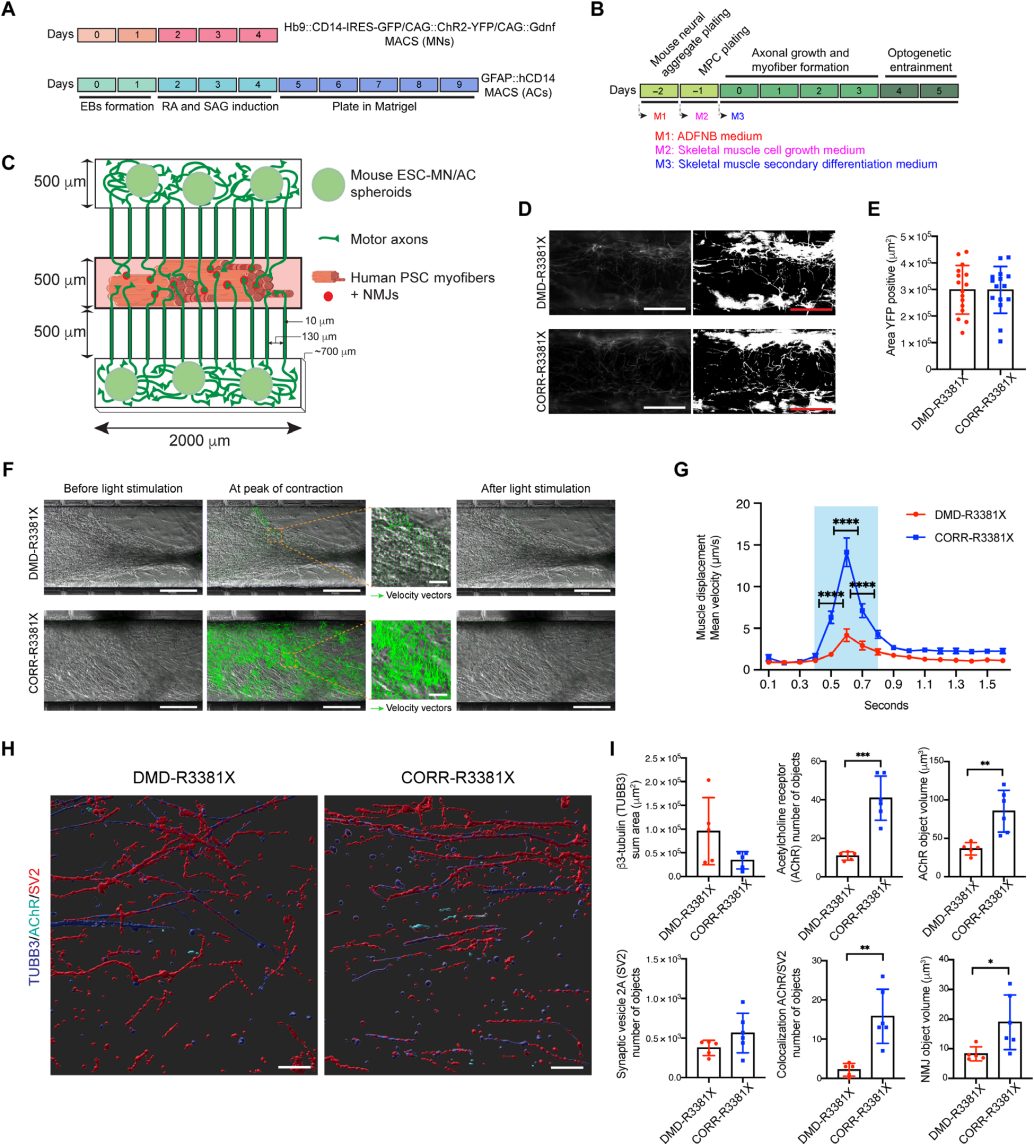
Optogenetic modeling of neuromuscular circuits in DMD with CRISPR-corrected isogenic controls. (**A**) Timeline of mouse ESC-MN and ESC-AC differentiation. RA, retinoic acid; SAG, smoothened agonist. (**B**) Timeline of the neuromuscular circuit assembly and medium changes in the microdevices. (**C**) Schematic of the microdevice. MPCs were plated in the central compartment. The two outer compartments held MN/AC spheroids. Motor axons projected through microchannels to innervate myofibers. (**D**) Representative images of YFP-positive motor axons in central compartments of microdevices containing MPCs at 0 hours and converted grayscale images. Scale bars, 250 μm. (**E**) Quantification of YFP-positive area in central compartments. *n* = 16. Values are means ± SEM; unpaired *t* test. (**F**) Particle image velocimetry (PIV) analysis in central compartments containing DMD- and CORR-R3381X neuromuscular circuits following optogenetic stimulation on day 5. Representative images show before, during, and after optogenetic stimulation. Green arrows represent velocity vectors. Scale bars, 250 μm. (**G**) Quantification of mean velocity in the central compartments containing DMD- and CORR-R3381X myofibers in response to optogenetic stimulation. Blue shading indicates the time during optogenetic stimulation. *n* = 24. Values are means ± SEM; two-way ANOVA and Sidak’s multiple comparisons test, ****P < 0.0001. (**H**) Representative 3D reconstruction images of immunocytochemistry of TUBB3 (blue), SV2 (red), and acetylcholine receptor (AChR; turquoise) in central compartments containing innervated DMD- and CORR-R3381X myofibers. Scale bars, 50 μm. (**I**) Quantification of TUBB3, SV2, AChR, and NMJs in central compartments containing DMD- and CORR-R3381X neuromuscular circuits. *n* = 5 for DMD-R3381X and *n* = 6 for CORR-R3381X. Values are means ± SD; unpaired *t* test, *P < 0.05, **P < 0.01, and ***P < 0.001.

Our GSEA results prompted us to investigate the effects of dystrophin-deficient myofibers on neuromuscular circuit formation and function. To model muscle-nerve connectivity in vitro with resemblance of anatomical separation in vivo, we used a compartmentalized microdevice to culture wild-type (WT) mouse embryonic stem cell (ESC)–derived MNs and the isogenic pair of human DMD-R3381X and CORR-R3381X MPC-derived myofibers. The microdevices were fabricated as previously described with slight modifications (*18*). We first generated spinal-type MNs expressing photosensor channelrhodopsin-2^H134R^ (ChR2-MNs) (*28*) and CAG::Gdnf transgenic astrocytes (ACs) from mouse ESCs (*29*) in separate cultures, followed by enrichment using magnetic-activated cell sorting (MACS) of both cell types and aggregation into neural spheroids (Fig. 3A) (*18*). On day −2, three spheroids of MACS-enriched ChR2-MNs and ACs were seeded into both outer compartments of the microdevice in a fibrin/Matrigel hydrogel (Fig. 3, B and C). On day −1, the isogenic pair of human DMD-R3381X and CORR-R3381X MPCs were loaded separately into the central compartment of the microdevice and then sealed in a fibrin/Matrigel hydrogel. The motor axons of mouse ESC–derived ChR2-MNs [labeled by yellow fluorescent protein (YFP)] grew through the microchannels from the outer compartments to the central compartment of the microdevice. On day 0, the initial distribution of ChR2-MN axons was similar in the microdevices containing DMD-R3381X and CORR-R3381X MPCs (Fig. 3D), as demonstrated by the quantification of YFP-positive area representing motor axons in the central compartment (Fig. 3, D and E). On day 0, the isogenic pair of human DMD-R3381X and CORR-R3381X MPCs were induced to form a 3D sheet of myofibers by switching to secondary differentiation medium (Fig. 3B). Contrary to CORR-R3381X myofibers, we noticed that DMD-R3381X myofibers were not evenly distributed nor mostly aligned with the long axis of the central compartment (Fig. 3F), which may reflect the abnormal gene regulation of skeletal muscle differentiation in DMD (Fig. 2I). In the following days, motor axons of mouse ESC–derived ChR2-MNs formed synaptic contacts with the human PSC–derived myofibers.

### Optogenetic modeling of neuromuscular circuits reveals compromised myofiber contraction and NMJ defects in DMD

Machado *et al.* (*18*) demonstrated that neural activity promotes NMJ formation in vitro; therefore, we entrained the light-responsive ChR2-MNs on days 4 and 5 for 1 hour/day after initiation of secondary differentiation (Fig. 3B). Blue light (470 nm) stimulation of mouse ChR2-MNs on day 5 induced neuron activation, which rapidly caused contraction of human MPC–derived myofibers in the central compartments, demonstrating the functionality of the newly formed neuromuscular circuits (movies S1 and S2). We quantified the velocity of myofiber contraction using particle image velocimetry (PIV), which measures the displacement of blocks of pixels between two consecutive images, frame by frame (*30*). In response to light stimulation, myofiber contraction and relaxation was visualized as velocity vectors with different sizes proportional to the local displacement (Fig. 3F). Compared to CORR-R3381X myofibers in microdevices, DMD-R3381X myofibers had fewer and smaller velocity vectors when stimulated by optogenetic activation of ChR2-MNs, indicating limited displacement in DMD conditions (Fig. 3F). Quantification of myofiber contraction velocity revealed a significantly reduced maximum contraction velocity in DMD-R3381X myofibers (~5 μm/s), compared with CORR-R3381X myofibers (~15 μm/s) (Fig. 3G).

Our GSEA results revealed that *CHRNA1* [acetylcholine receptor (AChR) subunit alpha] and *DMD* are among the core enrichment genes in GO muscle cell cellular homeostasis, in which both *CHRNA1* and *DMD* were down-regulated in DMD-R3381X compared to CORR-R3381X myogenic cultures (fig. S3). We then investigated whether the NMJs might be affected in the absence of muscle dystrophin protein. Immunocytochemistry of the presynaptic marker synaptic vesicle glycoprotein 2A (SV2) and β3-tubulin (TUBB3) in the myofiber compartments (Fig. 3H) did not show statistically significant differences in the number of SV2 objects and motor axon distribution between DMD-R3381X and CORR-R3381X conditions (Fig. 3I). Quantification of the postsynaptic marker nicotinic AChR revealed a significantly reduced number of AChR objects in DMD-R3381X myofiber compartments compared to CORR-R3381X myofiber compartments (Fig. 3, H and I). Accordingly, the number of NMJs, as determined by the colocalization of presynaptic and postsynaptic markers (SV2 and AChR), was significantly lower in DMD-R3381X than in CORR-R3381X conditions (Fig. 3, H and I). We also found that AChR and NMJ volumes were significantly reduced in DMD-R3381X myofiber compartments (Fig. 3, H and I). Together, our results suggest that NMJ defects in DMD may contribute to compromised myofiber contraction (Fig. 3G), thereby reflecting muscle weakness in patients with DMD.

### Pharmacological inhibition of TGFβ signaling in DMD-R3381X neuromuscular circuits rescues myofiber contraction phenotypes

**Fig. 4.**
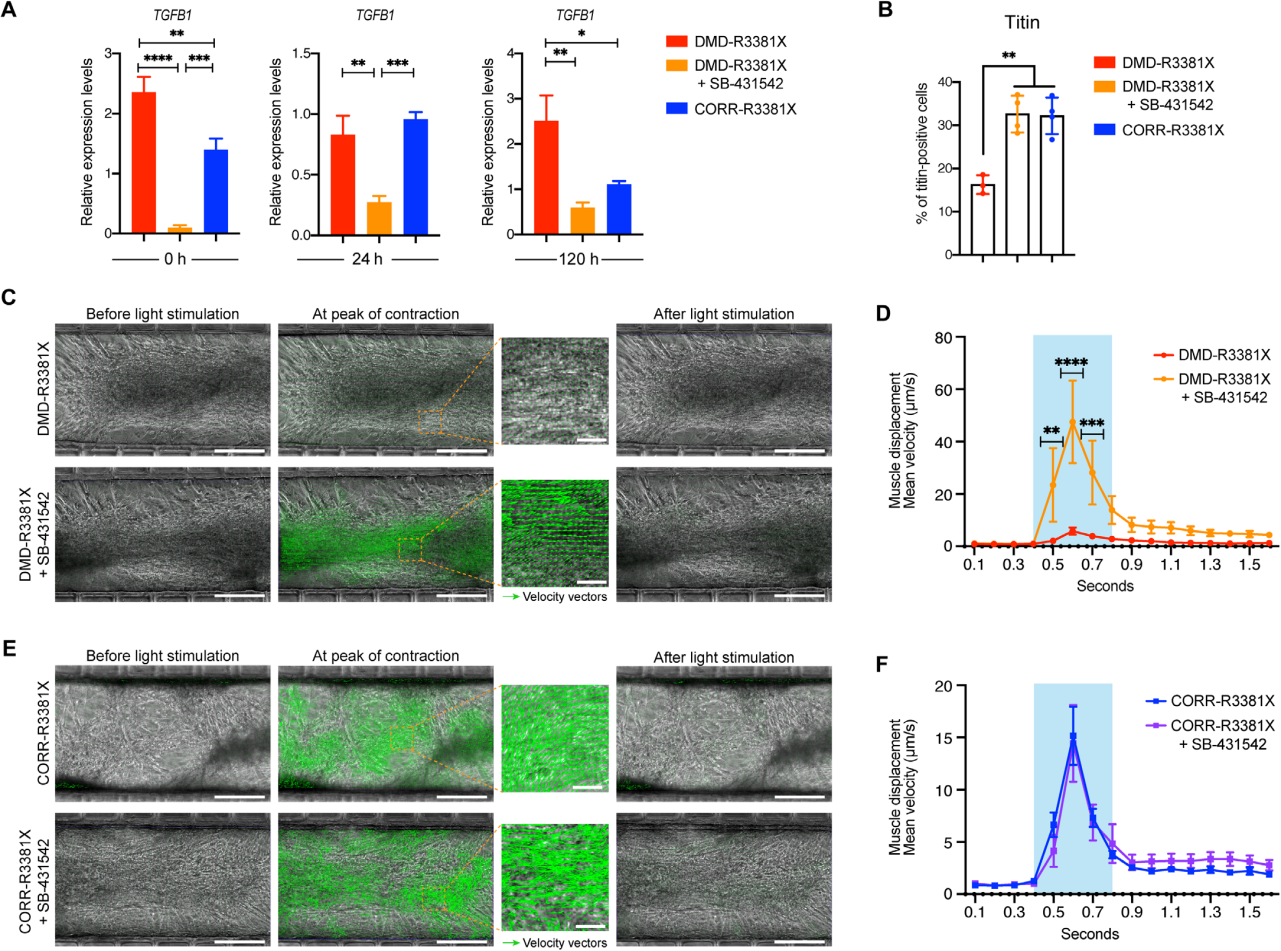
Inhibition of TGFβ signaling in DMD-R3381X neuromuscular circuits rescues their myofiber contractility defect. (**A**) Relative expression levels of *TGFB1* transcripts in 2D myogenic cultures of DMD-R3381X, DMD-R3381X + SB-431542, and CORR-R3381X at 0, 24, and 120 hours of secondary differentiation. *n* = 6. Values are means ± SEM. One-way ANOVA and Tukey’s multiple comparisons test, *P < 0.05, **P < 0.01, ***P < 0.001, and ****P < 0.0001. (**B**) Quantification of titin-positive cells in 2D myogenic cultures of DMD-R3381X, DMD-R3381X + SB-431542, and CORR-R3381X at 120 hours of secondary differentiation (representative images in fig. S4C). *n* = 4. Values are means ± SD. One-way ANOVA and Tukey’s multiple comparisons test, **P < 0.01. (**C**) PIV analysis in central compartments containing DMD-R3381X neuromuscular circuits not treated and treated with SB-431542 on day 5. Scale bars, 250 μm. (**D**) Quantification of mean velocity of DMD-R3381X myofibers not treated and treated with SB-431542 in response to optogenetic stimulation. *n* = 12. Values are means ± SEM. Two-way ANOVA and Sidak’s multiple comparisons test, **P < 0.01, ***P < 0.001, and ****P < 0.0001. (**E**) PIV analysis in central compartments containing CORR-R3381X neuromuscular circuits not treated and treated with SB-431542 on day 5. Scale bars, 250 μm. (**F**) Quantification of mean velocity of CORR-R3381X myofibers not treated and treated with SB-431542 in response to optogenetic stimulation. *n* = 12. Values are means ± SEM. Two-way ANOVA and Sidak’s multiple comparisons test, no significant difference. (C and E) Representative images show before, during, and after optogenetic stimulation. Green arrows represent velocity vectors. (D and F) Blue shading indicates the time during optogenetic stimulation.

Studies have shown that TGFβ signaling is down-regulated during the course of fetal myogenesis (*31*) and that it becomes abnormally up-regulated in DMD (*17*). Our transcriptome analysis showed down-regulation of *TGFB1* expression during the course of secondary differentiation, yet *TGFB1* transcript levels in DMD-R3381X were significantly higher than CORR-R3381X at 0 and 24 hours (fig. S4A). Furthermore, GSEA results revealed that up-regulated *TGFB1* and genes involved in TGFβ signaling were among core enrichment genes in GO negative regulation of myoblast differentiation associated with DMD-R3381X myogenic transcriptomes (fig. S4B). We then performed quantitative gene expression analysis in 2D myogenic cultures (fig. S4C) and confirmed that DMD-R3381X had a significantly higher *TGFB1* expression than CORR-R3381X at 0 and 120 hours of secondary differentiation (Fig. 4A). Moreover, *TGFB1* expression levels in DMD 2D myogenic cultures treated with SB-431542 (a selective inhibitor of TGFβ signaling) were significantly reduced at each time point, compared with untreated DMD cultures (Fig. 4A). Inhibition of TGFβ signaling improved the myogenic differentiation competence of DMD-R3381X cultures to similar levels of CORR-R3381X cultures at 120 hours (Fig. 4B and fig. S4C).

To test whether the increased myogenic differentiation competence might lead to any functional improvement in myofiber contractility, we treated the isogenic pair of DMD-R3381X and CORR-R3381X neuromuscular circuits with SB-431542 in microdevices for 5 days. Notably, PIV analysis of light-stimulated myofiber contraction showed a statistically significant increase in muscle displacement velocity in DMD-R3381X neuromuscular circuits treated with SB-431542 (Fig. 4, C and D, and movies S3 and S4). In contrast, treatment of SB-431542 in CORR-R3381X neuromuscular circuits did not have an effect on muscle displacement velocity (Fig. 4, E and F, and movies S5 and S6). The pharmacological rescue of the contractility phenotypes in DMD-R3381X myofibers by SB-431542 exceeded the displacement velocity of CORR-R3381X myofibers (fig. S4D). These results suggest that our isogenic pair of optogenetic neuromuscular circuits can serve as a microphysiological platform to assess potential drug candidates for treating DMD muscle weakness.

### Human neuromuscular circuit cocultures provide a drug screening platform for DMD and confirm the beneficial effects of SB-431542

**Fig. 5.**
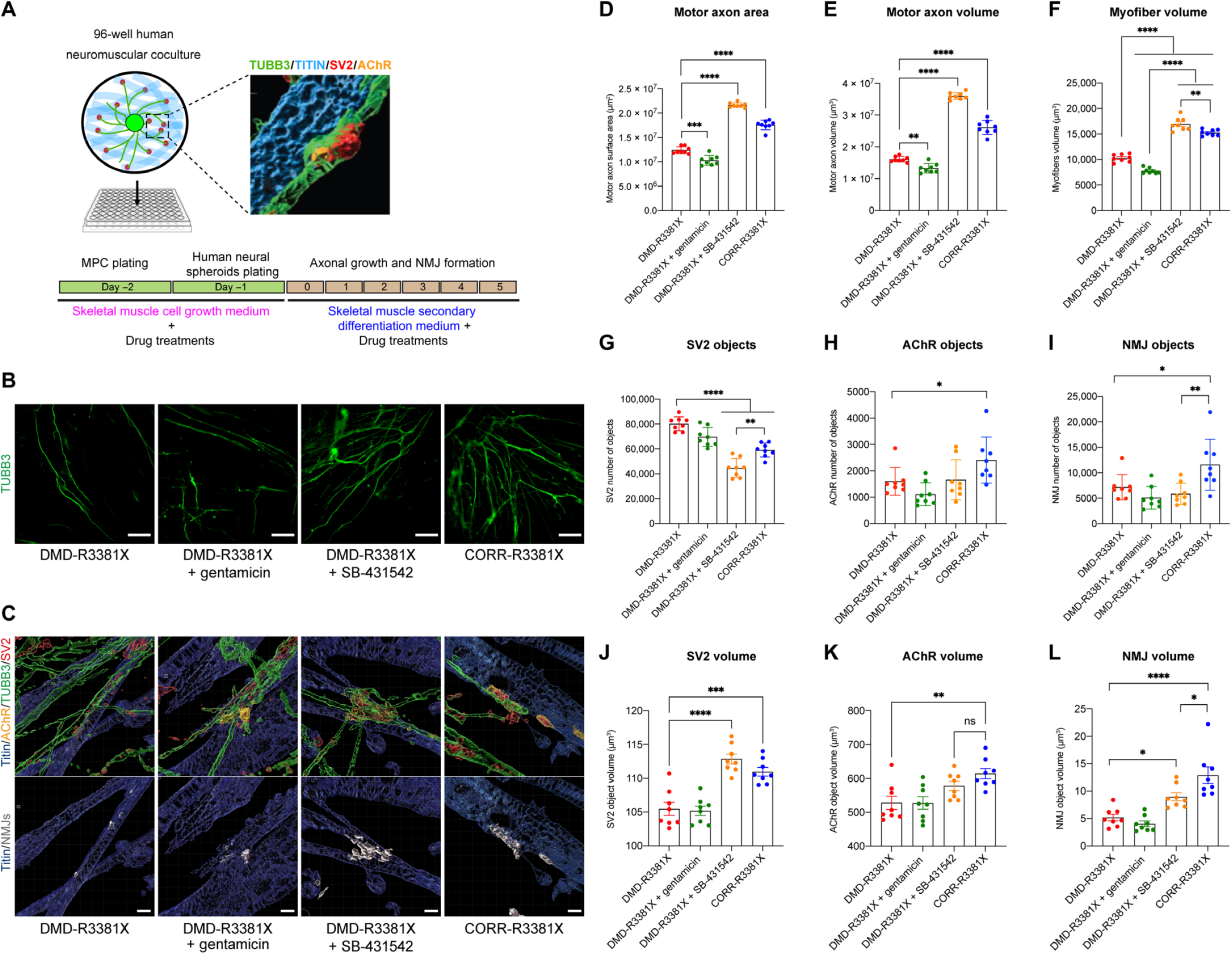
Human neuromuscular circuit cocultures provide a drug screening platform for DMD and confirm the beneficial effects of SB-431542. (**A**) Schematic of human neuromuscular circuit cocultures in a 96-well platform compatible with HCI imaging and analysis. Timeline of medium changes and pharmacological treatments are as indicated. (**B**) Representative images of motor axons projected from WT human ESC–derived MNs in DMD-R3381X, DMD-R3381X + gentamicin, DMD-R3381X + SB-431542, and CORR-R3381X neuromuscular circuit cocultures. Scale bars, 100 μm. (**C**) Representative 3D reconstruction images of human NMJs in DMD-R3381X, DMD-R3381X + gentamicin, DMD-R3381X + SB-431542, and CORR-R3381X neuromuscular circuit cocultures. Top: Titin in blue, the presynaptic markers TUBB3 in green and SV2 in red, and the postsynaptic marker AChR in orange. Bottom: NMJs, defined by colocalization of SV2 and AChR, were labeled in gray. Scale bars, 5 μm. (**D** to **L**) HCI analysis in human neuromuscular circuit cocultures of DMD-R3381X, DMD-R3381X + gentamicin, DMD-R3381X + SB-431542, and CORR-R3381X conditions, including quantification of motor axon surface area (D), motor axon volume (E), myofiber volume (F), number of SV2 objects (G), number of AChR objects (H), number of NMJ objects (I), SV2 volume (J), AChR volume (K), and NMJ volume (L). *n* = 8. Values are means ± SD for (D), (E), and (G) to (I) and means ± SEM for (F) and (J) to (L); one-way ANOVA, except (H), which was analyzed using *t* test, *P < 0.05, **P < 0.01, ***P < 0.001, and ****P < 0.0001. ns, not significant.

To better recapitulate human NMJ phenotypes and validate results from microdevices, we then developed a high-content imaging (HCI)–compatible, 96-well human neuromuscular circuit coculture assay amenable for drug screening and analysis of cellular phenotypes. We used WT human H9 ESC–derived MNs to form neuromuscular circuits with the isogenic pair of human PSC–derived myofibers. On day −2, we plated DMD-R3381X and CORR-R3381X human PSC–derived MPCs in flat-bottom Matrigel-coated 96-well plates. The day after, neural spheroids aggregated from MACS-sorted human ESC-MNs and mouse ESC-ACs were plated on top of the human PSC–derived MPCs into the center of each well (Fig. 5A). On day 0, the human neuromuscular cocultures were switched to secondary differentiation medium with and without drug treatments. Subsequently, human ESC–derived motor axons projected from the spheroids and innervated human PSC–derived myofibers. On day 5, the cocultures were fixed and stained with antibodies to myofiber, axon, presynaptic, and postsynaptic markers. Confocal images were acquired using a PerkinElmer Operetta HCI system, followed by multiparameter 3D image analysis.

As a proof of concept for assessing drugs in the HCI human neuromuscular circuit coculture assay, we included drugs with different mechanisms of action alongside SB-431542. For example, gentamicin, an aminoglycoside antibiotic that reads through premature stop codons (*32*), restores dystrophin expression in vitro and in *mdx* mice carrying *Dmd* nonsense mutations (*33*, *34*). To identify the optimal concentration of gentamicin for inducing readthrough effects, we first tested a range of gentamicin concentrations to treat DMD-R3381X MPCs during secondary differentiation and detected dystrophin protein expression in some of the DMD-R3381X myotubes (fig. S5, A and B). Note that the estimated levels of restored dystrophin protein in gentamicin-treated cultures were significantly lower than those in CORR-R3381X myogenic cultures (~13.2%; fig. S5, A and B). In parallel, we also tested the non-aminoglycoside readthrough compound PTC124 (*35*). Unexpectedly, we could not detect any restoration of dystrophin protein expression in DMD-R3381X myogenic cultures treated with PTC124 in a range of concentrations (from 1 to 35 μM; fig. S5C), suggesting that the *DMD* c.10141C>T nonsense mutation may favor gentamicin over PTC124. For this reason, we did not include PTC124 in the HCI human neuromuscular circuit coculture assay.

Analysis of HCI data showed that motor axon surface area and volume and myofiber volume were significantly higher in DMD-R3381X + SB-431542 or CORR-R3381X conditions, compared to DMD-R3381X or DMD-R3381X + gentamicin conditions (Fig. 5, B and D to F). Quantification of presynaptic markers showed that DMD-R3381X or DMD-R3381X + gentamicin conditions have significantly higher number of SV2 objects than DMD-R3381X + SB-431542 or CORR-R3381X conditions (Fig. 5, C and G). In contrast, quantification of postsynaptic marker showed that CORR-R3381X had significantly higher number of AChR objects than DMD-R3381X (Fig. 5, C and H). Quantification of NMJ objects, as defined by colocalization of SV2 and AChR objects, showed that CORR-R3381X had significantly higher number of NMJ objects than all other conditions (Fig. 5, C and I).

Next, we investigated the volumes of SV2, AChR, and NMJ objects. DMD-R3381X + SB-431542 or CORR-R3381X conditions have significant larger SV2 volume than DMD-R3381X or DMD-R3381X + gentamicin conditions (Fig. 5, C and J). Similar trends were observed by quantification of AChR volume (Fig. 5, C and K). Last, NMJ volumes in DMD-R3381X + SB-431542 or CORR-R3381X conditions were significantly larger than DMD-R3381X or DMD-R3381X + gentamicin conditions (Fig. 5, C and L). Briefly, our HCI analysis indicated that treatment of SB-431542 in DMD-R3381X neuromuscular cocultures not only benefited myofibers but also had a positive effect on MNs and NMJs. Together, these results suggest that the HCI human neuromuscular circuit coculture assay provides a useful platform for assessing drug efficacy and validates the beneficial effects of SB-431542 for ameliorating neuromuscular defects in DMD.

### Dysregulated genes affecting NMJ assembly and axon guidance respond to inhibition of TGFβ signaling in DMD myogenic cultures

**Fig. 6.**
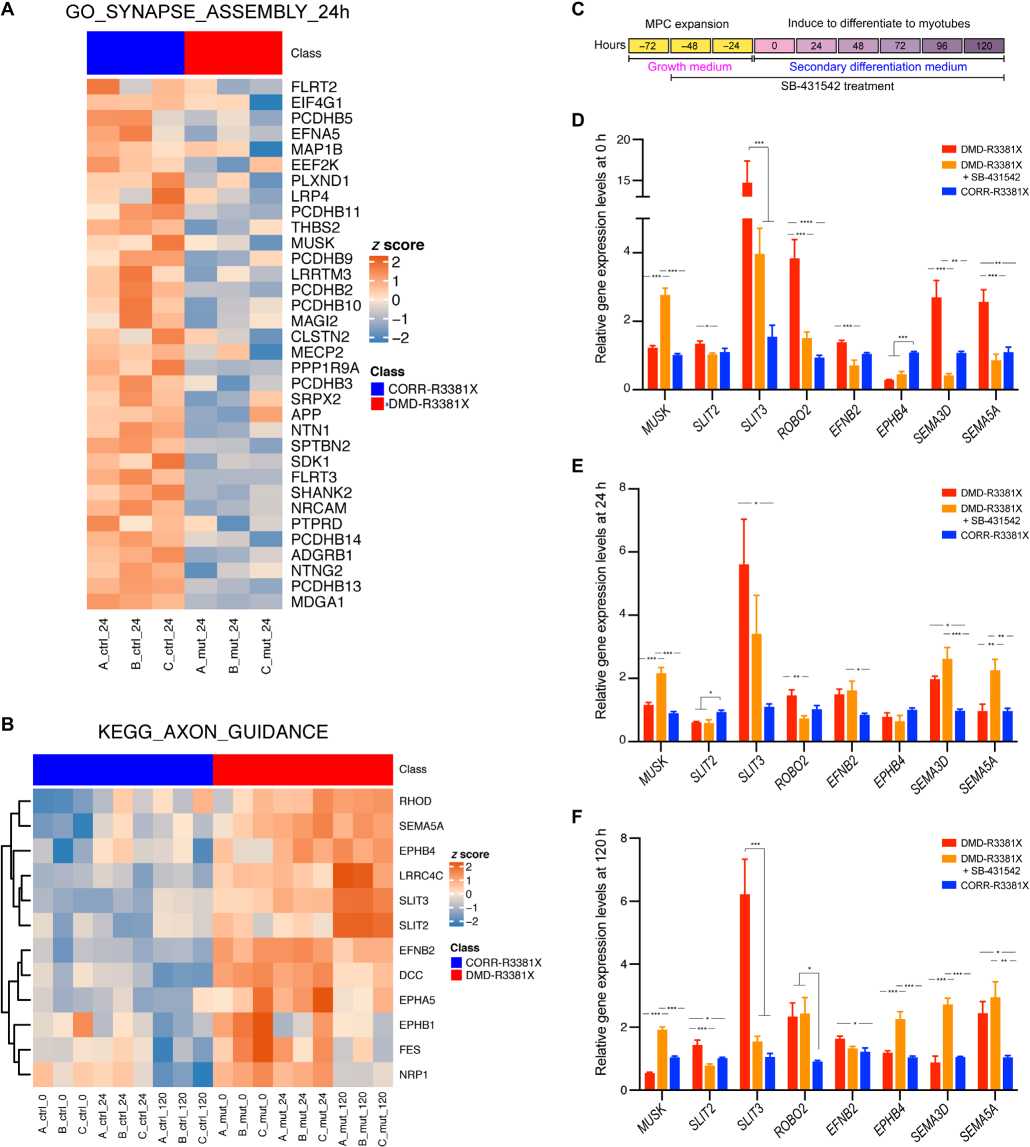
Aberrantly expressed genes in DMD-R3881X myogenic cultures respond to SB-431542 treatment. (**A**) Heatmap illustrating log_2_RPKM gene expression (row *z* scores) of core enrichment genes for GO synapse assembly at 24 hours; columns represent samples, and rows represent genes. (**B**) Heatmap illustrating log_2_RPKM gene expression (row *z* scores) of core enrichment genes for KEGG axon guidance observed across all three time points; columns represent samples, and rows represent genes. Row clustering was performed on the basis of Euclidean distance matrix and complete clustering. (**C**) A schematic timeline indicates treatment of SB-431542 in DMD-R3381X myogenic cultures with specified medium changes, followed by sample collection at 0, 24, and120 hours of secondary differentiation. (**D** to **F**) Relative expression levels of *MUSK*, *SLIT2*, *SLIT3*, *ROBO2*, *EFNB2*, *EPHB4*, *SEMA3D*, and *SEMA5A* in DMD-R3381X, DMD-R3381X + SB-431542, and CORR-R3381X myogenic cultures at 0 (D), 24 (E), and 120 hours (F) of secondary differentiation. For DMD-R3381X + SB-431542 cultures, DMD-R3381X MPCs were treated with SB-431542 in growth medium for 2 days before switching to secondary differentiation medium with SB-431542. *n* = 6. Values are means ± SEM. One-way ANOVA and Tukey’s multiple comparisons test, *P < 0.05, **P < 0.01, ***P < 0.001, and ****P < 0.0001.

Next, we sought to further elucidate possible mechanisms by which treatment of SB-431542 could ameliorate neuromuscular phenotypes in DMD. On the basis of the GSEA results (Fig. 2I), we identified core enrichment genes in GO synapse assembly, which were down-regulated in DMD-R3381X myogenic culture at 24 hours (Fig. 6A). In addition, we plotted core enrichment genes in KEGG axon guidance, which were up-regulated in DMD-R3381X myogenic cultures at all three time points (Fig. 6B and fig. S6). We hypothesized that treatment of SB-431542 may have effects on the expression of genes aberrantly up- or down-regulated in DMD-R3381X myogenic cultures. To test this, we cultured DMD-R3381X MPCs in growth medium with SB-431542 for 48 hours (i.e., 0 hours of secondary differentiation), followed by switching to secondary differentiation medium with SB-431542 for 24 and 120 hours (Fig. 6C). Untreated DMD-R3381X and CORR-R3381X myogenic cultures at the same time points were used for comparison. Among the core enrichment genes, we selected *MUSK* from GO synapse assembly and *SLIT2*, *SLIT3*, *ROBO2*, *EFNB2*, *EPHB4*, *SEMA3D*, and *SEMA5A* from KEGG axon guidance for analysis.

We then performed gene expression analysis of the selected core enrichment genes by qPCR in aneural myogenic cultures of DMD-R3381X, DMD-R3381X treated with SB-431542, and CORR-R3381X at 0, 24, and 120 hours of secondary differentiation (Fig. 6, D to F). Broadly speaking, we found that differences of gene expression levels between DMD-R3381X and CORR-R3381X were mostly consistent with the differentially expressed genes identified by transcriptome analysis. We found that *MUSK* expression was significantly increased in DMD-R3381X treated with SB-431542 at all three time points, compared to DMD-R3381X or CORR-R3381X (Fig. 6, D to F). *MUSK* encodes muscle-specific kinase, which plays a pivotal role in regulation of AChR clustering and NMJ formation (*36*). Thus, the qPCR results are consistent with increased AChR and NMJ volume in DMD-R3381X human neuromuscular circuits treated with SB-431542 (Fig. 5, K and L). Together, these results suggest that up-regulation of *MUSK* by inhibition of TGFβ signaling may promote NMJ assembly in DMD.

In contrast to *MUSK*, many core enrichment genes in axon guidance showed opposing trends in DMD-R3381X myogenic cultures treated with SB-431542. For example, *SLIT3* expression was significantly decreased to levels of the CORR-R3381X myogenic cultures at the three time points (Fig. 6, D to F). Similarly, there was a significant reduction in *SLIT2* expression at 0- and 120-hour time points (Fig. 6, D and F). *ROBO2* expression was significantly decreased at 0- and 24-hour time points (Fig. 6, D and E). Last, expression levels of *EFNB2* (ephrin), *SEMA3D*, and *SEMA5A* (semaphorins) were significantly down-regulated at 0 hours (Fig. 6D). *SLIT2* and *SLIT3* encode Slit family proteins, which interact with Robo proteins, are best known for regulating axon guidance as repulsive cues (*37*). Furthermore, studies suggest that ephrins and semaphorins are regulators of synapse formation (*38*). By and large, the qPCR results support increased motor axon surface area and volume in DMD-R3381X neuromuscular circuits treated with SB-431542 (Fig. 5, D and E). Together, these findings suggest that inhibition of TGFβ signaling normalizes many of the dysregulated axon guidance genes in DMD muscle, leading to a beneficial effect for NMJ assembly.

## DISCUSSION

In this study, we have developed the first optogenetic model of neuromuscular circuits for DMD with CRISPR-corrected isogenic controls by culturing human PSC–derived myofibers with mouse ESC–derived ChR2-MN/AC spheroids in a compartmentalized microdevice. Functional connectivity in this microphysiological model is demonstrated by optogenetic stimulation of ChR2-MN activity that specifically induces myofiber contraction. By comparing to CRISPR-corrected isogenic controls, the microphysiological model of DMD has enabled us to investigate mechanisms underlying muscle weakness and assess potential drugs for ameliorating pathophysiological hallmarks. To complement compartmentalized microdevices, we have established an HCI-compatible 96-well human neuromuscular circuit coculture assay amenable for high-throughput drug screening and quantitative analysis of cellular phenotypes.

Previous studies of DMD patient-derived PSC models are compared to unrelated WT derivatives or gene-edited controls by in-frame deletions to restore expression of shortened dystrophin proteins (*17*, *39*). Both experimental approaches have limitations in terms of disease modeling because (i) differences of genetic backgrounds between individuals may influence phenotypic variability and (ii) in-frame deletions only produce partially functional dystrophin proteins that likely elicit incomplete rescue of pathophysiology. In contrast, our genome editing strategy precisely corrects the *DMD* c.10141C>T mutation and restores full-length dystrophin protein expression under the control of endogenous promoter. Thus, our CRISPR-corrected isogenic control circumvents the aforementioned limitations and can serve as a benchmark for evaluating potential therapeutic strategies for DMD.

While most of our knowledge about NMJ alterations and muscle weakness in DMD is inferred from *mdx* mouse models, only few studies have reported abnormal electrophysiological properties and reduced synaptic fold size in patients with DMD (*11*, *12*). By modeling neuromuscular circuits in vitro, our isogenic pair of human PSC–derived myofibers cultured with optogenetic-controllable ChR2-MNs in a compartmentalized device recapitulates muscle-nerve connectivity resembling the anatomical arrangement of the MNs and myofibers in vivo. In response to light stimulation, we show that ChR2-MN activity–induced DMD-R3381X myofiber contraction has significantly reduced displacement velocity, compared to CORR-R3381X isogenic control myofibers. In addition, we show that compromised myofiber contraction is associated with a reduction of AChR and NMJ volumes in DMD-R3381X myofibers. In agreement with previous work (*11*, *12*), our results suggest that human skeletal muscle lacking dystrophin affects synaptic homeostasis of the NMJs.

To gain insights into the mechanisms underlying DMD pathophysiology, our analysis of myogenic transcriptomes and gene sets from the Canonical Pathways and Gene Ontology Biological Process has identified genes that show statistically significant, concordant differences between DMD and CRISPR-corrected isogenic controls, such as genes involved in regulating skeletal muscle differentiation and homeostasis, as well as axon guidance and NMJ assembly. Specifically, analysis of core enrichment genes in these gene sets shows that *TGFB1*, *SLIT2*, and *SLIT3* are up-regulated, while *CHRNA1*, *MUSK*, and *LRP4* are down-regulated in DMD myogenic transcriptomes. Consistent with our findings, multiple studies have shown evidence of TGFβ signaling pathway in skeletal muscle homeostasis and as a strong genetic modifier for DMD (*40*, *41*). In addition, studies have demonstrated that the Agrin-Lrp4-Musk signaling plays a critical role for NMJ formation and maturation and promotes AChR clustering (*36*). Furthermore, *Musk* transcript and protein levels are significantly decreased in *mdx* mice (*42*). Postnatal inactivation of Musk in mice results in loss of AChRs, disassembly of NMJs, and retraction of innervating motor axons (*43*). Last, augmenting expression of Musk in *mdx* mice by adeno-associated virus protects dystrophin-deficient muscle from contraction-induced injury, enhances the expression of utrophin (a homolog of dystrophin) and the DGC component β-dystroglycan, and ameliorates the NMJ morphology (*44*). Here, we show that pharmacological inhibition of TGFβ signaling in DMD neuromuscular circuits remarkably rescues the DMD myofiber contraction phenotypes and partially restores AChR and NMJ volumes. These functional corrections are associated with significant up-regulation of *MUSK* in DMD myofibers as demonstrated by inhibition of TGFβ signaling with SB-431542 in aneural myogenic cultures of DMD. Note that amelioration of NMJ defects in DMD neuromuscular circuits treated with SB-431542 cannot fully explain the remarkable rescue of myofiber contractility, suggesting that additional mechanisms may be involved. Studies have shown that TGFβ signaling negatively correlates with actin cytoskeletal remodeling to regulate muscle cell fusion (*45*) and that cytoskeletal reorganization is involved in AChR redistribution and anchoring (*46*). The contraction of skeletal muscle is mediated by release of Ca^2+^ from the sarcoplasmic reticulum into the cytoplasm. Dysregulation of Ca^2+^ handling and reactive oxygen species (ROS) production are pathological features of DMD (*47*). In addition to cytoskeletal remodeling, we speculate that Ca^2+^ signaling and ROS production in DMD may be altered in the presence of SB-431542. Future studies of signaling pathways in NMJ assembly, Ca^2+^ handling, and their cross-talk will further elucidate molecular mechanisms governing NMJ homeostasis and muscle contraction.

Apart from *MUSK*, many of the abnormally expressed axon guidance genes in DMD myogenic cultures are normalized by inhibition of TGFβ signaling with SB-431542, such as *SLIT2*, *SLIT3*, *ROBO2*, *EFNB2*, *SEMA3D*, and *SEMA5A*. This is reflected by the increased motor axon surface area and volume. While Slit-Robo signaling has been extensively studied for mediating axon repulsion, accumulating evidence suggests that guidance molecules play important roles in regulating synapse formation and plasticity (*38*). The core enrichment genes in KEGG axon guidance include genes encoding guidance molecules that are membrane-bound or secreted proteins, such as ephrins, semaphorins, netrin, and slits. On the basis of the transcriptomic profiling of DMD myogenic cultures, we presume that the secretome of DMD muscle may affect the NMJ homeostasis and function. We envisage a model of the early pathogenesis of DMD in vivo that is exacerbated by cycles of communications from myofibers to MNs and back at the NMJs during the disease progression. It will be of interest to investigate effects of DMD muscle–derived factors on neuromuscular connectivity between CRISPR-corrected PSC-derived myofibers and MN/AC spheroids treated with conditioned medium from DMD myogenic cultures.

Consistent with our transcriptome analysis, a recent study by Mournetas *et al.* (*48*) also identified down-regulation of genes involved in NMJ formation in DMD patient PSC-derived myotubes (e.g., *CHRNA1*) and DMD patient-derived primary myoblasts (e.g., *CHRNA1* and *MUSK*), compared to non-isogenic WT controls. Mournetas *et al.* used myoblasts from three DMD patients with in-frame duplication exons 3 to 26, out-of-frame deletion exons 8 to 43, or stop exon 7 c mutations, all of which disrupt the Dp427 but not the Dp71 isoform, whereas the DMD-R3381X mutation in our study affects both isoforms. This suggests that the aberrant gene expression affecting NMJ assembly may be primarily due to loss of Dp427, although we cannot exclude additional contributions from loss of Dp71. To address this, it will be of interests to compare DMD myogenic transcriptomes in the presence and absence of Dp71. In contrast to our study and others (*17*), up-regulation of TGFβ signaling in DMD PSC–derived myogenic cultures was not detected by Mournetas *et al.* This may be explained by variation between genetic backgrounds and differences in myogenic differentiation protocols and media (*24*, *49*). Note that the myogenic differentiation performed by Mournetas *et al.* was from human PSCs (day 0) to myotubes (day 25), and the cell culture media contain inhibitors of TGFβ signaling from days 0 to 17, whereas our differentiation medium did not. Together, future comparative studies of DMD myogenic transcriptomes and validation with experiments will further elucidate common underlying mechanisms in DMD skeletal muscle.

As previously described in patients with DMD and *mdx* mice (*33*, *34*), DMD-R3381X myogenic cultures treated with gentamicin can restore some levels of dystrophin protein expression, but it is incomparable to restoration achieved by precise genome editing. It has been suggested that 20% of endogenous levels of dystrophin uniformly distributed may be sufficient to prevent disease progression (*50*) and ~20 to 50% of dystrophin levels are required to normalize the NMJ abnormalities of *mdx* mice (*51*). In agreement with previous studies, our HCI human neuromuscular circuit coculture assay does not show significant improvements on DMD neuromuscular circuits treated with gentamicin. Nevertheless, a high-throughput screening of large compound libraries in the HCI platform with human neuromuscular circuits will allow us to identify novel drugs capable of improving NMJ assembly and muscle function.

In an effort to avoid the toxicity associated with the antibiotic, gentamicin has been replaced by other readthrough compounds, such as PTC124 (*52*). Paradoxically, a recent multicenter, randomized, double-blind, phase 3 trial has concluded that there is no significant difference between patients treated with ataluren (PTC124) and placebo group; nevertheless, a significant effect of ataluren is recorded in prespecified subgroup of patients (*53*). In our hands, we did not detect restored dystrophin protein expression in DMD-R3381X myogenic cultures treated with PTC124 from low (1 μM) to high (35 μM) contractions, suggesting that the effect of PTC124 may be allele specific. Collectively, these results suggest that future studies integrating pharmacogenomic research are required to further elucidate the mechanisms by which PTC124 can achieve nonsense mutation readthrough. With additional isogenic pairs of DMD and CRISPR-corrected isogenic controls, microphysiological models of human neuromuscular circuits developed in this study can facilitate assessing the efficacy and toxicity of potential drug candidates at the preclinical stage of therapeutic development, leading to personalized medicine for patients with DMD.

Future studies can implement several modifications to further improve the physiological relevance, reproducibility, and stability of our neuromuscular circuit model. Currently, we use MNs and ACs derived from WT mouse or unrelated healthy human ESCs. To further elucidate the roles of DGC at the NMJs and a variety of synapses in the nervous systems, it will be ideal to generate MNs, ACs, and skeletal muscle from the same isogenic pair of DMD and CRISPR-corrected human PSCs for culturing in the compartmentalized microdevice or 96-well coculture platform. Note that the approach of using isogenic, multilineage differentiation from human PSCs is applicable to other neuromuscular disorders, such as ALS, spinal muscular atrophy, and myasthenia gravis. The direct measurement of myofiber contraction by PIV will allow the assessment of functional phenotypes in models for these conditions, in addition to quantifying axonal, myofiber, and NMJ morphology. Last, our current system does not sustain long-term cultures of neuromuscular circuits under conditions that strengthen myofibers (e.g., inhibition of TGFβ signaling) as it leads to culture collapse. We intend to improve myofiber stability in the next generation of microdevices. Recent advances in biomaterials for tissue engineering can generate aligned nanofibrillar and supportive extracellular matrix–like scaffold capable of stabilizing myofiber contraction in long-term cultures (*54*).

In summary, optogenetic modeling of human neuromuscular circuits in DMD with CRISPR-corrected isogenic controls warrants a microphysiological model recapitulating in vivo NMJ pathophysiology, which contributes to muscle weakness in patients with DMD. Beyond modulating skeletal muscle homeostasis, pharmacological inhibition of TGFβ signaling can ameliorate the NMJ defects and remarkably rescue myofiber contractility in DMD. Our results suggest that targeting pathways in neuromuscular connectivity may be an effective therapeutic strategy for treating DMD regardless of types of mutations.

## MATERIALS AND METHODS

### Generation and maintenance of DMD patient-derived PSCs

We obtained the fibroblast line from a patient with DMD carrying the *DMD* c.10141C>T (p.R3381X) mutation from the MRC Centre for Neuromuscular Diseases Biobank with informed consent under appropriate ethical approval by Hammersmith and Queen Charlotte’s and Chelsea Hospital (REC reference 06/Q0406/33) and by NRES (National Research Ethics Service) Committee London-Stanmore (REC reference 13/LO/1826; IRAS project ID: 141100). We used a six factor–based reprogramming protocol and a recently developed EPSCM (*21*, *22*) to generate DMD patient-specific PSCs, referred to as DMD-R3381X ePSCs. In vitro differentiation of human ePSCs and analysis were performed as previously described (*23*).

### Immunocytochemistry

Routine immunostaining protocol for human PSCs, MPCs, and myotubes were preceded by cell fixation with 4% paraformaldehyde (Santa Cruz Biotechnology, sc-281692) for 20 min at room temperature (RT). Before and after fixation, the cells were washed three times, 5 min each, with 1× phosphate-buffered saline (PBS). For intracellular markers, the cells were permeabilized with 0.5% Triton X-100 (Sigma-Aldrich, T8787) in 1× PBS for 15 min at RT. After three washes of 5 min in 1× PBS, they were blocked with 10% goat serum (Sigma-Aldrich, G9023) in 1× PBS for 1 hour at RT, and then, primary antibodies were incubated overnight at 4°C in blocking buffer at the appropriate dilution (table S2). The day after, the cells were washed with 1× PBS three times for 5 min each time.

Appropriate Alexa Fluor secondary antibodies (table S3) and 4′,6-diamidino-2-phenylindole (1:1000; Sigma-Aldrich, D9542) were incubated for 1 hour at RT in blocking buffer. The cells were kept in 1× PBS at 4°C in the dark until analysis. Image acquisition was performed with IN Cell Analyzer 2200 and IN Cell Analyzer 6000 from GE Healthcare Life Science, a confocal microscope (LSM 800), and Operetta CLS High-Content Analysis System.

For quantitative analysis of the images, three technical replicates with eight images per replicate were analyzed. Automated image analysis was done with Developer Toolbox from GE Healthcare Life Sciences. The quantification protocols were based on a number of defined targets and the parameters that we wanted to measure in those targets. To define a target as positive or negative, the intensity threshold was established on the basis of the pixel intensity of the negative control condition (no primary antibody incubation). In addition, object size limits were set up when looking at nuclear markers. Fusion index is defined by the percentage of nuclei within cells positive for MF20 marker containing three or more than three nuclei.

### RNA extraction

Total RNA was extracted from at least 5 × 10^5^ cells using RNeasy Plus Mini Kit (QIAGEN, 74134) following the manufacturer’s instructions and eluted in 10 μl of deoxyribonuclease/ribonuclease-free water. The concentration was determined using the Qubit RNA HS Assay Kit (Invitrogen, Q32852) and the Qubit Fluorometer according to the company’s protocol.

### Real-time qPCR

The High-Capacity cDNA Reverse Transcription Kit (Applied Biosystems, 4368814) was used to convert mRNA into cDNA. Three hundred nanograms of mRNA in up to 10 μl were mixed with 2× reverse transcription master mix following the manufacturer’s instructions. PowerUp SYBR Green Master Mix (Applied Biosystems, A25742) was used for real-time qPCR. The reaction was set up as per the manufacturer’s instructions, and the primers are specified in table S4. Cycle thresholds were produced by the StepOne Plus software (Applied Biosystems) and normalized to the values of the reference gene *ACTB* (*55*), and relative gene expression levels were expressed as fold changes.

### Karyotyping analysis

Multiplex fluorescent in situ hybridization karyotype analysis was performed on human ePSC lines as explained in (*56*) with a minor change. Human ePSCs were grown in knockout Dulbecco’s modified Eagle’s medium (DMEM) supplemented with 15% fetal bovine serum, 1× glutamine-penicillin-streptomycin, 1× nonessential amino acids, and human recombinant leukemia inhibitory factor (1 ng/ml) for 24 hours followed by 10 μM Y-27632 dihydrochloride (Tocris, 1254) treatment for 2 to 3 hours before metaphase harvesting.

### CRISPR-Cas9–mediated gene correction

To construct the donor targeting vectors, we used Gibson Assembly (New England BioLabs). The 1-kb left and right homology arms were PCR-amplified from the parental *DMD* PSCs. At the same time, the point mutation c.10141C>T was corrected with a specific primer for each (table S4). The pMCS-AAT_PB-PGKpuroTK (*57*) plasmid was used as a template to amplify the *piggyBac* selection cassette (PGK-puro∆tk) and the backbone of the vectors (table S5). The four PCR fragments were joined together by their 40–base pair (bp) overlapping ends in a Gibson Assembly reaction. The recombinant Cas9 protein was a gift from N. Geijsen (*58*). The single guide RNA (sgRNA) was synthesized by Synthego. The Cas9/sgRNA ribonucleoprotein complex and donor targeting vector were incubated together with the cells and electroporated using Lonza Nucleofector 4D device (Lonza, AAF-1002B).

### Myogenic differentiation from human PSCs

To generate MPCs and myotubes from human PSCs, we used a transgene-free myogenic differentiation protocol developed by Chal *et al.* (*24*). Briefly, the PSCs were plated on Matrigel-coated plates and induced to differentiate into the myogenic lineage for 3 to 4 weeks. The primary differentiation culture was expanded, enriched in Skeletal Muscle Cell Growth Medium (PromoCell, C-23060), and cryopreserved. To generate myotubes, the human ePSC–derived MPCs were induced to undergo secondary differentiation by culturing in secondary differentiation medium containing DMEM/F12 + glutamine (Invitrogen, 11320-033), 1% insulin-transferrin-selenium (Gibco, 414000459), 1% N2 supplement (Gibco, 17502048), 0.2% penicillin-streptomycin (10,000 U/ml), and 1% l-glutamine (Gibco, 25030149) (*24*).

### Pharmacological treatments of MPCs during secondary differentiation

MPCs were expanded in Skeletal Muscle Cell Growth Medium until reaching the desired number of cells. The pharmacological treatments started at the last 2 days in Skeletal Muscle Cell Growth Medium and continued during the 5 days of secondary differentiation. MPCs were treated with 10 μM SB-431542 (Tocris, 1614) as in (*31*). After testing a range of gentamicin (Sigma-Aldrich, G1397) concentrations (10 to 600 μM), 200 μM gentamicin was used in the 96-well human neuromuscular circuit coculture assay, as 600 μM gentamicin caused cytotoxicity. PTC124 (Generon, A8553) was tested in a range of concentrations (1, 5, 10, 17, 25, and 35 μM).

### Immunoblot analysis

Human PSC–derived myotubes were harvested and lysed with radioimmunoprecipitation assay buffer supplemented with a cocktail of protease inhibitors. Primary human myoblast–derived myotubes were used as a positive control [MRC CMMD (Medical Research Council Centre for Neuromuscular Diseases) Biobank ID: 8206]. NuPAGE Novex 3 to 8% tris-acetate gel (Invitrogen, EA0375BOX) was used to resolve the proteins, followed by their transfer with the Trans-Blot Turbo Transfer System (Bio-Rad) into a nitrocellulose membrane (Bio-Rad). The membrane was blocked for 1 hour with Odyssey block solution (LI-COR Bioscience, 927-50000) and probed with dystrophin (1:750; Fisher Scientific, PA5-32388), vinculin (1:1000; Sigma-Aldrich, MAB3574), and β-actin (1:5000; Sigma-Aldrich, A5316) primary antibodies for 2 hours at RT. After three washes with 1× PBS containing 0.1% Tween 20 for 15 min each, the membrane was incubated with biotinylated anti-rabbit secondary antibody (1:1000) for 2 hours, followed by IRDye 800CW Donkey Anti-Rabbit IgG (H + L) (1:10,000; LI-COR Biosciences, 926-32213) and IRDye 680RD Goat Anti-Mouse IgG (H + L) (1:10000, LI-COR Biosciences, 926-68070) for 1 hour at RT. Signals were visualized and acquired by the Odyssey CLx Infrared Imaging System (LICOR Biosciences) using Image Studio acquisition software.

### 3D compartmentalized muscle-nerve cultures

Hb9::CD14-IRES-GFP/CAG::ChR2-YFP and GFAP::hCD14/CAG::Gdnf transgenic mouse ESC clones were generated as described in (*18*, *29*). Mouse ESCs of both genotypes were dissociated with 0.25% trypsin (Gibco, 25200056) and plated in suspension nontreated culture dishes (Corning, CLS430591) to grow them as floating EBs for 5 days in ADFNB medium containing Advanced DMEM/F-12 and Neurobasal medium at 1:1 ratio, 1% of penicillin-streptomycin (10,000 U/ml), 1% l-glutamine, 50 μM β-mercaptoethanol, 2% of MACS NeuroBrew-21 with vitamin A (Miltenyi Biotec, 130-093-566), 1% of N2 supplement, and 0.1% bovine serum albumin. The medium was supplemented with 1 μM retinoic acid (Sigma-Aldrich, R2625) and 0.5 μM smoothened agonist (Merck, SML1314) for the last 3 days of culture. EBs were then dissociated, and ESC-derived MNs were isolated by anti-CD14 MACS enrichment as previously described in (*59*). GFAP::hCD14 ESCs were differentiated to EBs with the same protocol, and then, EBs were replated into T75 flasks coated with Growth Factor Reduced (GFR)–Matrigel diluted 1:50 in DMEM and cultured in ADFNB medium for seven more days. The adherent EBs were dissociated with 0.25% trypsin, and ESC-derived ACs were isolated by anti-CD14 MACS enrichment as previously described in (*29*). To generate MN/AC neural spheroids, 5 × 10^3^ mouse ESC–derived MACS-sorted ChR2-MNs along with 5 × 10^3^ mouse ESC–derived MACS-sorted ACs (*18*, *29*, *59*) were plated in 96-well round-bottom Lipidure-coated plates for 24 hours in ADFNB medium.

3D microdevices were manufactured as described (*18*) with minor changes. The devices were designed with one central compartment and two outer compartments linked with microchannels (*18*). Briefly, soft lithography with polydimethylsiloxane (PDMS) was used to fabricate the devices (*60*). Plastic bottom dishes (diameter, 35 mm; ibidi, 81156) were coated with a thin layer of NOA-73 resin using a cell scraper and partially ultraviolet (UV)–cured for 10 s at 55 J/cm^2^. Then, the PDMS arrays were placed on the resin layer (microgrooves down) and UV-cured with same conditions but for 1 min. Before seeding cells, the devices were UV-sterilized and coated with GFR-Matrigel diluted 1:100 in DMEM in a vacuum. Three MN/AC spheroids were plated in each outer compartment of the microdevices and then immobilized with thrombin in a fibrin/Matrigel hydrogel. Twenty-four hours after, human PSC–derived MPCs were plated in the central compartment and then sealed in a fibrin/Matrigel hydrogel. Twenty-four hours later, MPCs were induced to differentiate into myotubes by changing medium to secondary differentiation medium. On days 4 and 5 of differentiating muscle cells, the cultures were optically entrained for 1 hour a day with blue light (450-nm wavelength) at 5 Hz, 20-ms epoch, and 40% light-emitting diode (LED) intensity in dishes placed on top of a custom-built heat sink and LED assembly (*18*, *61*, *62*). Royal-Blue Luxeon Rebel LEDs mounted to a 20-mm Star Base were purchased from Quadica Developments Inc. (Lethbridge, Canada). The light intensity of the LEDs was adjusted so as to cause reliable activation of channelrhodopsin-2 while limiting any potential damage caused by long-term exposure to high-energy light. The culture medium was supplemented with Antioxidant Supplement (1×; Sigma-Aldrich, A1345).

#### Imaris image visualization and analysis

3D reconstructions of neuromuscular cocultures were made using the Bitplane Imaris 9.1.2 software. Synapses were labeled with antibodies against synaptic vesicle glycoprotein 2A (SV2) and nicotinic AChR. Furthermore, myofibers and motor axons were labeled with antibodies against titin and TUBB3, respectively. NMJs were defined by constructing a colocalization channel for SV2 and AChR using the Imaris coloc plug-in. Subsequently, 3D reconstructions for each channel of *Z*-stack images were made using the surface function in Imaris. From these reconstructions, the total number and morphology (volume and surface area) of individual SV2, AChR, and colocalized objects was analyzed, as well as total TUBB3 area for total axon coverage.

### Human MN differentiation

Human H9 ESCs (WiCell) carrying an Hb9::CD14 transgene inserted into the AAVS1 locus were cultured on laminin-521–coated plates in StemMACS iPS-Brew XF medium (Miltenyi Biotec, 130-104-368). On day 4, colonies were carefully harvested and seeded into Matrigel-coated plates in StemMACS iPS-Brew XF medium. When colonies attached, the medium was changed to MN progenitors (MNP) basal differentiation medium containing 50% of DMEM/F12, 50% of Neurobasal medium (Gibco, 21103049), 1% of N2, 2% of MACS NeuroBrew-21 (Miltenyi Biotech, 130-093-566), and 0.1% of penicillin-streptomycin-glutamine (100×; Gibco, 10378-016) supplemented with 3 μM CHIR99021, 2 μM SB-431542, and 0.2 μM LDN193189 and cultured in this medium for 5 days. On day 6, the cells were dissociated and seeded into Matrigel-coated dishes in the same medium supplemented with 0.1 μM RA (retinoic acid) and 0.5 μM purmorphamine and kept in the same medium for six more days. On day 13, the cells were dissociated as single cells and plated at 5 × 10^5^ cells/cm^2^ in suspension in MNP medium supplemented with 0.5 μM RA and 0.1 μM purmorphamine. Seven days later, the immature MNs were dissociated, and we proceeded to do MACS sorting to enrich the population. I.L. has approval from the UK Stem Cell Bank steering committee (no. SCS11-06) to import human H9 ESCs.

### Optogenetic stimulation of microdevice cultures and video analysis

We recorded 2-s bright-field videos (10 frames per second) of microdevice cocultures with an inverted fluorescence microscope (Olympus X73). Optogenetic stimulation was carried by illumination of the microdevice cultures for 500 ms at 100% LED intensity with an optical fiber–coupled 470-nm LED light source (Thorlabs, M470F3) controlled by a LED driver (Thorlabs, DC2200). The light guide was positioned 1 cm above the microdevice, and the light intensity at this distance was 0.2 mW/mm^2^, which is sufficient to trigger action potentials in ChR2-expressing neurons (*63*). Myofiber contraction velocity was measured as area displacement between successive video frames by PIV with the PIVlab package (*30*) within MATLAB (MathWorks) as described in (*18*).

### Neuromuscular cocultures in a 96-well format

Human ePSC-MPCs were plated as a confluent monolayer in 96-well plate at a density of 20,000 per well (Fig. 5A). The next day, one spheroid aggregated from MACS-sorted Hb9::CD14 human ESC-MNs and mouse *CAG::Gdnf* ESC-ACs (5000 per cell type) (*18*) was placed into the center of each well on top of the developing myofibers, and the cocultures were grown for six more days. Then, the cells were fixed with 4% paraformaldehyde/15% sucrose for 5 min; washed five times with PBS; and immunostained for the antigens TUBB3, SV2, nicotinic AChR, and titin (see table S2). Images were acquired with a PerkinElmer Operetta CLS high-content analysis system to image the entire well using the 20× water objective, confocal mode, and a binning of 2. In total, 65,856 images were acquired, composed of seven Z stacks per field of view, set at 2-μm intervals with 5% overlap. Forty-nine fields of view were taken per well, and 48 wells were imaged in total. High-content image analysis was carried out with the Harmony 4.8 software (PerkinElmer). Briefly, images were analyzed in 3D, and 3D masks were created for each channel and filtered on the basis of intensity and morphology to remove background or staining artifacts. Total axon volume and surface area based on TUBB3 staining was calculated per well to quantify total axon outgrowth. The total number of individual myofiber objects, based on titin staining, was calculated per well along with the average morphological characteristics (volume/surface area/sphericity). The total number of SV2 and AChR objects was calculated per well along with average morphological characteristics (volume/surface area/sphericity). NMJ objects were defined by SV2 and AChR colocalization and calculated as total number per well along with average morphological characteristics (volume/surface area/sphericity).

### Transcriptome sequencing and analysis

Total RNA from 18 samples (three biological replicates of two genotypes at three time points) was processed by the Barts and the London Genome Centre at QMUL (Queen Mary University of London). Sequencing was performed on Illumina NextSeq 500, High Output run sequencer with 75-bp paired end and 14 million reads mean depth per sample.

Alignment to the reference genome GRCh38 was performed using HISAT2 v2.1.0 (*64*), and read count was performed using HTSeq v0.11.1 (*65*). For the analysis and interpretation of the data, only genes that achieved a minimum of one read count per million reads in at least four samples were kept. A total of 14,477 genes matched this condition, and 12,833 of them were protein-coding genes. Conditional quantile normalization was performed counting for gene length and GC content to remove technical variability in the data inherent to the sequencing approach (*66*), and a log_2_ transformed RPKM (reads per kilobase per million mapped reads) expression matrix was generated (*67*).

Differential expression analysis was performed using the limma R package v3.38.2 and voom normalization, using the linear model (*68*) on R v3.5.1. To consider experimental repeats, the duplicateCorrelation function was used. GSEA was performed for each comparison using the ranked t-statistic and the GSEA tool GenePattern (*69*) for Canonical Pathways and Gene Ontology Biological Process from the Molecular Signatures Database (c2.cp.v7.1 and c5.bp.v7.1). RNA sequencing data have been deposited to Gene Expression Omnibus under the accession number GSE159273.

## Supplementary Material

20210910-1

## Supplementary Materials

### This PDF file includes:

Figs. S1 to S6 Tables S1 to S5 Legends for movies S1 to S6 Legends for data files S1 and S2

### Other Supplementary Material for this manuscript includes the following:

Data files S1 and S2

Correction (21 October 2022):
In the Supplementary Materials PDF, the scale on the y-axis in Figure S4D was corrected to 20, 40, 60, 80 (μm/s). The original version is available here:

## Notes

View/request a protocol for this paper from *Bio-protocol*.

## References

[1] J. R. Mendell, C. Shilling, N. D. Leslie, K. M. Flanigan, R. Al-Dahhak, J. Gastier-Foster, K. Kneile, D. M. Dunn, B. Duval, A. Aoyagi, C. Hamil, M. Mahmoud, K. Roush, L. Bird, C. Rankin, H. Lilly, N. Street, R. Chandrasekar, R. B. Weiss, Evidence-based path to newborn screening for Duchenne muscular dystrophy. Ann. Neurol. 71, 304–313 (2012). 22451200 10.1002/ana.23528

[2] T. A. Rando, The dystrophin-glycoprotein complex, cellular signaling, and the regulation of cell survival in the muscular dystrophies. Muscle Nerve 24, 1575–1594 (2001). 11745966 10.1002/mus.1192

[3] K. Bushby, R. Finkel, D. J. Birnkrant, L. E. Case, P. R. Clemens, L. Cripe, A. Kaul, K. Kinnett, C. McDonald, S. Pandya, J. Poysky, F. Shapiro, J. Tomezsko, C. Constantin, Diagnosis and management of Duchenne muscular dystrophy, part 1: Diagnosis, and pharmacological and psychosocial management. Lancet Neurol. 9, 77–93 (2010). 19945913 10.1016/S1474-4422(09)70271-6

[4] C. Angelini, E. Peterle, Old and new therapeutic developments in steroid treatment in Duchenne muscular dystrophy. Acta Myol. 31, 9–15 (2012). 22655511 PMC3440806

[5] K. Bushby, R. Finkel, B. Wong, R. Barohn, C. Campbell, G. P. Comi, A. M. Connolly, J. W. Day, K. M. Flanigan, N. Goemans, K. J. Jones, E. Mercuri, R. Quinlivan, J. B. Renfroe, B. Russman, M. M. Ryan, M. Tulinius, T. Voit, S. A. Moore, H. L. Sweeney, R. T. Abresch, K. L. Coleman, M. Eagle, J. Florence, E. Gappmaier, A. M. Glanzman, E. Henricson, J. Barth, G. L. Elfring, A. Reha, R. J. Spiegel, M. W. O’donnell, S. W. Peltz, C. M. Mcdonald; PTC124-GD-007-DMD STUDY GROUP, Ataluren treatment of patients with nonsense mutation dystrophinopathy. Muscle Nerve 50, 477–487 (2014). 25042182 10.1002/mus.24332 PMC4241581

[6] K. Dzierlega, T. Yokota, Optimization of antisense-mediated exon skipping for Duchenne muscular dystrophy. Gene Ther. 27, 407–416 (2020). 32483212 10.1038/s41434-020-0156-6

[7] A.-F. E. Schneider, A. Aartsma-Rus, Developments in reading frame restoring therapy approaches for Duchenne muscular dystrophy. Expert Opin. Biol. Ther. 21, 343–359 (2021). 33074029 10.1080/14712598.2021.1832462

[8] G. S. K. Pilgram, S. Potikanond, R. A. Baines, L. G. Fradkin, J. N. Noordermeer, The roles of the dystrophin-associated glycoprotein complex at the synapse. Mol. Neurobiol. 41, 1–21 (2010). 19899002 10.1007/s12035-009-8089-5 PMC2840664

[9] F. Jerusalem, A. G. Engel, M. R. Gomez, Duchenne dystrophy. II. Morphometric study of motor end-plate fine structure. Brain 97, 123–130 (1974). 4434165 10.1093/brain/97.1.123

[10] E. M. van der Pijl, M. van Putten, E. H. Niks, J. J. G. M. Verschuuren, A. Aartsma-Rus, J. J. Plomp, Characterization of neuromuscular synapse function abnormalities in multiple Duchenne muscular dystrophy mouse models. Eur. J. Neurosci. 43, 1623–1635 (2016). 27037492 10.1111/ejn.13249

[11] R. M. Lovering, S. R. Iyer, B. Edwards, K. E. Davies, Alterations of neuromuscular junctions in Duchenne muscular dystrophy. Neurosci. Lett. 737, 135304 (2020). 32818587 10.1016/j.neulet.2020.135304 PMC7541569

[12] S. Y. Ng, V. Ljubicic, Recent insights into neuromuscular junction biology in Duchenne muscular dystrophy: Impacts, challenges, and opportunities. EBioMedicine 61, 103032 (2020). 33039707 10.1016/j.ebiom.2020.103032 PMC7648118

[13] J. W. McGreevy, C. H. Hakim, M. A. McIntosh, D. Duan, Animal models of Duchenne muscular dystrophy: From basic mechanisms to gene therapy. Dis. Model. Mech. 8, 195–213 (2015). 25740330 10.1242/dmm.018424 PMC4348559

[14] R. A. Jones, C. Harrison, S. L. Eaton, M. Llavero Hurtado, L. C. Graham, L. Alkhammash, O. A. Oladiran, A. Gale, D. J. Lamont, H. Simpson, M. W. Simmen, C. Soeller, T. M. Wishart, T. H. Gillingwater, Cellular and molecular anatomy of the human neuromuscular junction. Cell Rep. 21, 2348–2356 (2017). 29186674 10.1016/j.celrep.2017.11.008 PMC5723673

[15] A. Paredes-Redondo, Y. Lin, Human induced pluripotent stem cells: Challenges and opportunities in developing new therapies for muscular dystrophies, in *eLS* (John Wiley & Sons, 2019).

[16] J. Chal, O. Pourquié, Making muscle: Skeletal myogenesis in vivo and in vitro. Development 144, 2104–2122 (2017). 28634270 10.1242/dev.151035

[17] I. Y. Choi, H. Lim, K. Estrellas, J. Mula, T. V. Cohen, Y. Zhang, C. J. Donnelly, J.-P. Richard, Y. J. Kim, H. Kim, Y. Kazuki, M. Oshimura, H. L. Li, A. Hotta, J. Rothstein, N. Maragakis, K. R. Wagner, G. Lee, Concordant but varied phenotypes among duchenne muscular dystrophy patient-specific myoblasts derived using a human iPSC-based model. Cell Rep. 15, 2301–2312 (2016). 27239027 10.1016/j.celrep.2016.05.016

[18] C. B. Machado, P. Pluchon, P. Harley, M. Rigby, V. G. Sabater, D. C. Stevenson, S. Hynes, A. Lowe, J. Burrone, V. Viasnoff, I. Lieberam, In vitro modeling of nerve–muscle connectivity in a compartmentalized tissue culture device. Adv. Biosyst. 3, 1800307 (2019). 31428672 10.1002/adbi.201800307 PMC6699992

[19] T. Osaki, S. G. M. Uzel, R. D. Kamm, Microphysiological 3D model of amyotrophic lateral sclerosis (ALS) from human iPS-derived muscle cells and optogenetic motor neurons. Sci. Adv. 4, eaat5847 (2018). 30324134 10.1126/sciadv.aat5847 PMC6179377

[20] E. S. Boyden, F. Zhang, E. Bamberg, G. Nagel, K. Deisseroth, Millisecond-timescale, genetically targeted optical control of neural activity. Nat. Neurosci. 8, 1263–1268 (2005). 16116447 10.1038/nn1525

[21] J. Yang, D. J. Ryan, W. Wang, J. C.-H. Tsang, G. Lan, H. Masaki, X. Gao, L. Antunes, Y. Yu, Z. Zhu, J. Wang, A. A. Kolodziejczyk, L. S. Campos, C. Wang, F. Yang, Z. Zhong, B. Fu, M. A. Eckersley-Maslin, M. Woods, Y. Tanaka, X. Chen, A. C. Wilkinson, J. Bussell, J. White, R. Ramirez-Solis, W. Reik, B. Göttgens, S. A. Teichmann, P. P. L. Tam, H. Nakauchi, X. Zou, L. Lu, P. Liu, Establishment of mouse expanded potential stem cells. Nature 550, 393–397 (2017). 29019987 10.1038/nature24052 PMC5890884

[22] A. C. Wilkinson, D. J. Ryan, I. Kucinski, W. Wang, J. Yang, S. Nestorowa, E. Diamanti, J. C.-H. Tsang, J. Wang, L. S. Campos, F. Yang, B. Fu, N. Wilson, P. Liu, B. Gottgens, Expanded potential stem cell media as a tool to study human developmental hematopoiesis in vitro. Exp. Hematol. 76, 1–12.e5 (2019). 31326613 10.1016/j.exphem.2019.07.003 PMC6859476

[23] J. Kim, B. Lana, S. Torelli, D. Ryan, F. Catapano, P. Ala, C. Luft, E. Stevens, E. Konstantinidis, S. Louzada, B. Fu, A. Paredes-Redondo, A. E. Chan, F. Yang, D. L. Stemple, P. Liu, R. Ketteler, D. L. Selwood, F. Muntoni, Y. Lin, A new patient-derived iPSC model for dystroglycanopathies validates a compound that increases glycosylation of α-dystroglycan. EMBO Rep. 20, e47967 (2019). 31566294 10.15252/embr.201947967 PMC6832011

[24] J. Chal, Z. Al Tanoury, M. Hestin, B. Gobert, S. Aivio, A. Hick, T. Cherrier, A. P. Nesmith, K. K. Parker, O. Pourquié, Generation of human muscle fibers and satellite-like cells from human pluripotent stem cells in vitro. Nat. Protoc. 11, 1833–1850 (2016). 27583644 10.1038/nprot.2016.110

[25] T. Kawaguchi, E. Niba, A. Rani, Y. Onishi, M. Koizumi, H. Awano, M. Matsumoto, M. Nagai, S. Yoshida, S. Sakakibara, N. Maeda, O. Sato, H. Nishio, M. Matsuo, Detection of dystrophin Dp71 in human skeletal muscle using an automated capillary western assay system. Int. J. Mol. Sci. 19, 1546 (2018). 29789502 10.3390/ijms19061546 PMC6032138

[26] M. Farea, A. Q. M. Rani, K. Maeta, H. Nishio, M. Matsuo, Dystrophin Dp71ab is monoclonally expressed in human satellite cells and enhances proliferation of myoblast cells. Sci. Rep. 10, 17123 (2020). 33051488 10.1038/s41598-020-74157-y PMC7553993

[27] R. García-Rodríguez, M. Hiller, L. Jiménez-Gracia, Z. van der Pal, J. Balog, K. Adamzek, A. Aartsma-Rus, P. Spitali, Premature termination codons in the DMD gene cause reduced local mRNA synthesis. Proc. Natl. Acad. Sci. U.S.A. 117, 16456–16464 (2020). 32616572 10.1073/pnas.1910456117 PMC7368324

[28] G. Nagel, M. Brauner, J. F. Liewald, N. Adeishvili, E. Bamberg, A. Gottschalk, Light activation of channelrhodopsin-2 in excitable cells of *Caenorhabditis elegans* triggers rapid behavioral responses. Curr. Biol. 15, 2279–2284 (2005). 16360690 10.1016/j.cub.2005.11.032

[29] J. B. Bryson, C. B. Machado, M. Crossley, D. Stevenson, V. Bros-Facer, J. Burrone, L. Greensmith, I. Lieberam, Optical control of muscle function by transplantation of stem cell–derived motor neurons in mice. Science 344, 94–97 (2014). 24700859 10.1126/science.1248523 PMC5947756

[30] W. Thielicke, E. J. Stamhuis, PIVlab—Towards user-friendly, affordable and accurate digital particle image velocimetry in MATLAB. J. Open Res. Softw. 2, e30 (2014).

[31] M. R. Hicks, J. Hiserodt, K. Paras, W. Fujiwara, A. Eskin, M. Jan, H. Xi, C. S. Young, D. Evseenko, S. F. Nelson, M. J. Spencer, B. Van Handel, A. D. Pyle, ERBB3 and NGFR mark a distinct skeletal muscle progenitor cell in human development and hPSCs. Nat. Cell Biol. 20, 46–57 (2018). 29255171 10.1038/s41556-017-0010-2 PMC5962356

[32] T. Hermann, Aminoglycoside antibiotics: Old drugs and new therapeutic approaches. Cell. Mol. Life Sci. 64, 1841–1852 (2007). 17447006 10.1007/s00018-007-7034-x PMC11136281

[33] V. Malik, L. R. Rodino-Klapac, L. Viollet, J. R. Mendell, Aminoglycoside-induced mutation suppression (stop codon readthrough) as a therapeutic strategy for Duchenne muscular dystrophy. Ther. Adv. Neurol. Disord. 3, 379–389 (2010). 21179598 10.1177/1756285610388693 PMC3002642

[34] E. R. Barton-Davis, L. Cordier, D. I. Shoturma, S. E. Leland, H. L. Sweeney, Aminoglycoside antibiotics restore dystrophin function to skeletal muscles of mdx mice. J. Clin. Invest. 104, 375–381 (1999). 10449429 10.1172/JCI7866 PMC481050

[35] E. M. Welch, E. R. Barton, J. Zhuo, Y. Tomizawa, W. J. Friesen, P. Trifillis, S. Paushkin, M. Patel, C. R. Trotta, S. Hwang, R. G. Wilde, G. Karp, J. Takasugi, G. Chen, S. Jones, H. Ren, Y.-C. Moon, D. Corson, A. A. Turpoff, J. A. Campbell, M. M. Conn, A. Khan, N. G. Almstead, J. Hedrick, A. Mollin, N. Risher, M. Weetall, S. Yeh, A. A. Branstrom, J. M. Colacino, J. Babiak, W. D. Ju, S. Hirawat, V. J. Northcutt, L. L. Miller, P. Spatrick, F. He, M. Kawana, H. Feng, A. Jacobson, S. W. Peltz, H. L. Sweeney, PTC124 targets genetic disorders caused by nonsense mutations. Nature 447, 87–91 (2007). 17450125 10.1038/nature05756

[36] L. Li, W.-C. Xiong, L. Mei, Neuromuscular junction formation, aging, and disorders. Annu. Rev. Physiol. 80, 159–188 (2018). 29195055 10.1146/annurev-physiol-022516-034255

[37] K. Brose, K. S. Bland, K. H. Wang, D. Arnott, W. Henzel, C. S. Goodman, M. Tessier-Lavigne, T. Kidd, Slit proteins bind robo receptors and have an evolutionarily conserved role in repulsive axon guidance. Cell 96, 795–806 (1999). 10102268 10.1016/s0092-8674(00)80590-5

[38] K. Shen, C. W. Cowan, Guidance molecules in synapse formation and plasticity. Cold Spring Harb. Perspect. Biol. 2, a001842 (2010). 20452946 10.1101/cshperspect.a001842 PMC2845208

[39] C. S. Young, M. R. Hicks, N. V. Ermolova, H. Nakano, M. Jan, S. Younesi, S. Karumbayaram, C. Kumagai-Cresse, D. Wang, J. A. Zack, D. B. Kohn, A. Nakano, S. F. Nelson, M. C. Miceli, M. J. Spencer, A. D. Pyle, A single CRISPR-Cas9 deletion strategy that targets the majority of DMD patients restores dystrophin function in hiPSC-derived muscle cells. Cell Stem Cell 18, 533–540 (2016). 26877224 10.1016/j.stem.2016.01.021 PMC4826286

[40] E. Ceco, E. M. McNally, Modifying muscular dystrophy through transforming growth factor-β. FEBS J. 280, 4198–4209 (2013). 23551962 10.1111/febs.12266 PMC3731412

[41] T. N. Burks, R. D. Cohn, Role of TGF-β signaling in inherited and acquired myopathies. Skelet. Muscle 1, 19 (2011). 21798096 10.1186/2044-5040-1-19 PMC3156642

[42] S. J. P. Pratt, S. B. Shah, C. W. Ward, J. P. Kerr, J. P. Stains, R. M. Lovering, Recovery of altered neuromuscular junction morphology and muscle function in mdx mice after injury. Cell. Mol. Life Sci. 72, 153–164 (2015). 24947322 10.1007/s00018-014-1663-7 PMC4282693

[43] B. A. Hesser, O. Henschel, V. Witzemann, Synapse disassembly and formation of new synapses in postnatal muscle upon conditional inactivation of MuSK. Mol. Cell. Neurosci. 31, 470–480 (2006). 16337809 10.1016/j.mcn.2005.10.020

[44] S. Trajanovska, J. Ban, J. Huang, P. Gregorevic, M. Morsch, D. G. Allen, W. D. Phillips, Muscle specific kinase protects dystrophic mdx mouse muscles from eccentric contraction-induced loss of force-producing capacity. J. Physiol. 597, 4831–4850 (2019). 31340406 10.1113/JP277839

[45] F. Girardi, A. Taleb, M. Ebrahimi, A. Datye, D. G. Gamage, C. Peccate, L. Giordani, D. P. Millay, P. M. Gilbert, B. Cadot, F. Le Grand, TGFβ signaling curbs cell fusion and muscle regeneration. Nat. Commun. 12, 750 (2021). 33531466 10.1038/s41467-020-20289-8 PMC7854756

[46] H. Wu, W. C. Xiong, L. Mei, To build a synapse: Signaling pathways in neuromuscular junction assembly. Development 137, 1017–1033 (2010). 20215342 10.1242/dev.038711 PMC2835321

[47] D. G. Allen, N. P. Whitehead, S. C. Froehner, Absence of dystrophin disrupts skeletal muscle signaling: Roles of Ca^2+^, reactive oxygen species, and nitric oxide in the development of muscular dystrophy. Physiol. Rev. 96, 253–305 (2016). 26676145 10.1152/physrev.00007.2015 PMC4698395

[48] V. Mournetas, E. Massouridès, J.-B. Dupont, E. Kornobis, H. Polvèche, M. Jarrige, A. R. L. Dorval, M. R. F. Gosselin, A. Manousopoulou, S. D. Garbis, D. C. Górecki, C. Pinset, Myogenesis modelled by human pluripotent stem cells: A multi-omic study of Duchenne myopathy early onset. J. Cachexia. Sarcopenia Muscle 12, 209–232 (2021). 33586340 10.1002/jcsm.12665 PMC7890274

[49] L. Caron, D. Kher, K. L. Lee, R. McKernan, B. Dumevska, A. Hidalgo, J. Li, H. Yang, H. Main, G. Ferri, L. M. Petek, L. Poellinger, D. G. Miller, D. Gabellini, U. Schmidt, A human pluripotent stem cell model of facioscapulohumeral muscular dystrophy-affected skeletal muscles. Stem Cells Transl. Med. 5, 1145–1161 (2016). 27217344 10.5966/sctm.2015-0224 PMC4996435

[50] D. J. Wells, What is the level of dystrophin expression required for effective therapy of Duchenne muscular dystrophy? J. Muscle Res. Cell Motil. 40, 141–150 (2019). 31289969 10.1007/s10974-019-09535-9

[51] E. M. van der Pijl, M. van Putten, E. H. Niks, J. J. G. M. Verschuuren, A. Aartsma-Rus, J. J. Plomp, Low dystrophin levels are insufficient to normalize the neuromuscular synaptic abnormalities of mdx mice. Neuromuscul. Disord. 28, 427–442 (2018). 29631954 10.1016/j.nmd.2018.02.013

[52] M. Dabrowski, Z. Bukowy-Bieryllo, E. Zietkiewicz, Advances in therapeutic use of a drug-stimulated translational readthrough of premature termination codons. Mol. Med. 24, 25 (2018). 30134808 10.1186/s10020-018-0024-7 PMC6016875

[53] C. M. McDonald, C. Campbell, R. E. Torricelli, R. S. Finkel, K. M. Flanigan, N. Goemans, P. Heydemann, A. Kaminska, J. Kirschner, F. Muntoni, A. N. Osorio, U. Schara, T. Sejersen, P. B. Shieh, H. L. Sweeney, H. Topaloglu, M. Tulinius, J. J. Vilchez, T. Voit, B. Wong, G. Elfring, H. Kroger, X. Luo, J. M. Intosh, T. Ong, P. Riebling, M. Souza, R. J. Spiegel, S. W. Peltz, E. Mercuri; Clinical Evaluator Training Group; ACT DMD Study Group, Ataluren in patients with nonsense mutation Duchenne muscular dystrophy (ACT DMD): A multicentre, randomised, double-blind, placebo-controlled, phase 3 trial. Lancet 390, 1489–1498 (2017). 28728956 10.1016/S0140-6736(17)31611-2

[54] K. H. Nakayama, M. Quarta, P. Paine, C. Alcazar, I. Karakikes, V. Garcia, O. J. Abilez, N. S. Calvo, C. S. Simmons, T. A. Rando, N. F. Huang, Treatment of volumetric muscle loss in mice using nanofibrillar scaffolds enhances vascular organization and integration. Commun. Biol. 2, 170 (2019). 31098403 10.1038/s42003-019-0416-4 PMC6505043

[55] J. C. W. Hildyard, A. M. Finch, D. J. Wells, Identification of qPCR reference genes suitable for normalizing gene expression in the mdx mouse model of Duchenne muscular dystrophy. PLOS ONE 14, e0211384 (2019). 30699165 10.1371/journal.pone.0211384 PMC6353192

[56] C. A. Agu, F. A. C. Soares, A. Alderton, M. Patel, R. Ansari, S. Patel, S. Forrest, F. Yang, J. Lineham, L. Vallier, C. M. Kirton, Successful generation of human induced pluripotent stem cell lines from blood samples held at room temperature for up to 48 hr. Stem Cell Rep. 5, 660–671 (2015). 10.1016/j.stemcr.2015.08.012 PMC4624992 26388286

[57] K. Yusa, S. T. Rashid, H. Strick-Marchand, I. Varela, P. Q. Liu, D. E. Paschon, E. Miranda, A. Ordóñez, N. R. F. Hannan, F. J. Rouhani, S. Darche, G. Alexander, S. J. Marciniak, N. Fusaki, M. Hasegawa, M. C. Holmes, J. P. Di Santo, D. A. Lomas, A. Bradley, L. Vallier, Targeted gene correction of α1-antitrypsin deficiency in induced pluripotent stem cells. Nature 478, 391–394 (2011). 21993621 10.1038/nature10424 PMC3198846

[58] D. S. D’Astolfo, R. J. Pagliero, A. Pras, W. R. Karthaus, H. Clevers, V. Prasad, R. J. Lebbink, H. Rehmann, N. Geijsen, Efficient intracellular delivery of native proteins. Cell 161, 674–690 (2015). 25910214 10.1016/j.cell.2015.03.028

[59] C. B. Machado, K. C. Kanning, P. Kreis, D. Stevenson, M. Crossley, M. Nowak, M. Iacovino, M. Kyba, D. Chambers, E. Blanc, I. Lieberam, Reconstruction of phrenic neuron identity in embryonic stem cell-derived motor neurons. Development 141, 784–794 (2014). 24496616 10.1242/dev.097188 PMC3912827

[60] T. Masters, W. Engl, Z. L. Weng, B. Arasi, N. Gauthier, V. Viasnoff, Easy fabrication of thin membranes with through holes. Application to protein patterning. PLOS ONE 7, e44261 (2012). 22952944 10.1371/journal.pone.0044261 PMC3432078

[61] W. Wefelmeyer, D. Cattaert, J. Burrone, Activity-dependent mismatch between axo-axonic synapses and the axon initial segment controls neuronal output. Proc. Natl. Acad. Sci. U.S.A. 112, 9757–9762 (2015). 26195803 10.1073/pnas.1502902112 PMC4534224

[62] M. S. Grubb, J. Burrone, Activity-dependent relocation of the axon initial segment fine-tunes neuronal excitability. Nature 465, 1070–1074 (2010). 20543823 10.1038/nature09160 PMC3196626

[63] H. Wang, J. Peca, M. Matsuzaki, K. Matsuzaki, J. Noguchi, L. Qiu, D. Wang, F. Zhang, E. Boyden, K. Deisseroth, H. Kasai, W. C. Hall, G. Feng, G. J. Augustine, High-speed mapping of synaptic connectivity using photostimulation in channelrhodopsin-2 transgenic mice. Proc. Natl. Acad. Sci. 104, 8143–8148 (2007). 17483470 10.1073/pnas.0700384104 PMC1876585

[64] D. Kim, B. Langmead, S. L. Salzberg, HISAT: A fast spliced aligner with low memory requirements. Nat. Methods 12, 357–360 (2015). 25751142 10.1038/nmeth.3317 PMC4655817

[65] S. Anders, P. T. Pyl, W. Huber, HTSeq—A Python framework to work with high-throughput sequencing data. Bioinformatics 31, 166–169 (2015). 25260700 10.1093/bioinformatics/btu638 PMC4287950

[66] K. D. Hansen, R. A. Irizarry, Z. Wu, Removing technical variability in RNA-seq data using conditional quantile normalization. Biostatistics 13, 204–216 (2012). 22285995 10.1093/biostatistics/kxr054 PMC3297825

[67] A. Mortazavi, B. A. Williams, K. McCue, L. Schaeffer, B. Wold, Mapping and quantifying mammalian transcriptomes by RNA-Seq. Nat. Methods 5, 621–628 (2008). 18516045 10.1038/nmeth.1226 PMC13303166

[68] M. E. Ritchie, B. Phipson, D. Wu, Y. Hu, C. W. Law, W. Shi, G. K. Smyth, *limma* powers differential expression analyses for RNA-sequencing and microarray studies. Nucleic Acids Res. 43, e47 (2015). 25605792 10.1093/nar/gkv007 PMC4402510

[69] M. Reich, T. Liefeld, J. Gould, J. Lerner, P. Tamayo, J. P. Mesirov, GenePattern 2.0. Nat. Genet. 38, 500–501 (2006). 16642009 10.1038/ng0506-500

