# Supplementary material for: Optogenetic modeling of human neuromuscular circuits in Duchenne muscular dystrophy with CRISPR and pharmacological corrections
Source: Sci Adv. 2021 Sep 10;7(37):eabi8787. doi: 10.1126/sciadv.abi8787 (PMC8442926; doi:10.1126/sciadv.abi8787)
Supplement: Supplementary file 2 — Figs. S1 to S6 Tables S1 to S5 Legends for movies S1 to S6 Legends for data files S1 and S2 [file sciadv.abi8787_sm.v2.pdf]

CORRECTED 21 OCTOBER 2022; SEE ERRATUM

Science Advances
AAAS

Supplementary Materials for

Optogenetic modeling of human neuromuscular circuits in Duchenne muscular dystrophy with CRISPR and pharmacological corrections

Amaia Paredes-Redondo, Peter Harley, Eleni Maniati, David Ryan, Sandra Louzada, Jinhong Meng, Anna Kowala, Beiyuan Fu, Fengtang Yang, Pentao Liu, Silvia Marino, Olivier Pourquié, Francesco Muntoni, Jun Wang, Ivo Lieberam, Yung-Yao Lin*

*Corresponding author. Email: yy.lin@qmul.ac.uk

Published 10 September 2021, *Sci. Adv.* **7**, eabi8787 (2021)
DOI: 10.1126/sciadv.abi8787

The PDF file includes:

Figs. S1 to S6
Tables S1 to S5
Legends for movies S1 to S6
Legends for data files S1 and S2

Other Supplementary Material for this manuscript includes the following:

Movies S1 to S6
Data files S1 and S2

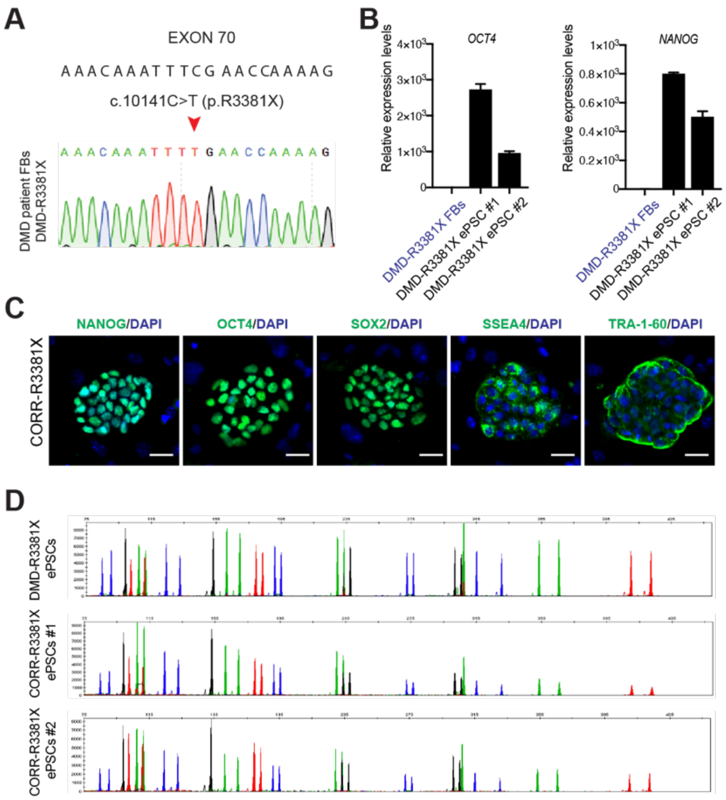

Fig. S1. Characterization of a pair of DMD patient-derived and isogenic control ePSCs.

(A) *DMD* c.10141C>T (p.R3381X) mutation confirmed by sequencing analysis in DMD patient's derived fibroblasts.

(B) Relative expression of *OCT4* and *NANOG* pluripotency markers in two independent DMD-R3381X ePSC clones. DMD fibroblasts did not express pluripotency genes. N=3, technical replicates, values are mean ± SD,

(C) Positive immunocytochemistry of NANOG, OCT4, SOX2, SSEA4 and TRA-1-60 in CORR-R3381X ePSCs. Scale bars are 100 μm.

(D) Microsatellite analysis confirmed common parental origin of the two independent CORR-R3381X ePSCs generated clones.

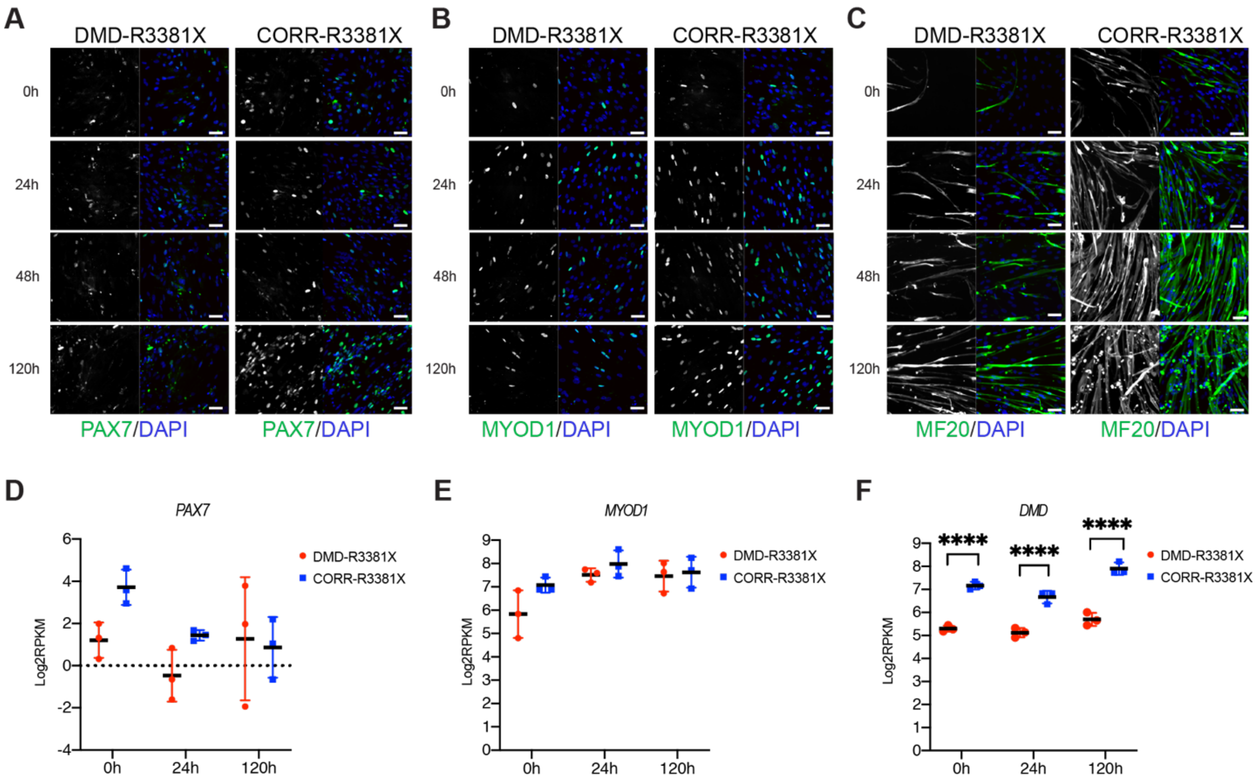

Fig. S2. Expression of myogenic markers in DMD- and CORR-R3381X MPCs and myotubes.

(A) Representative images of immunocytochemistry of PAX7 in DMD and CORR-R3381X MPCs and at 24, 48 and 120h in secondary differentiation medium. Scale bars are 50 µm.

(B) Representative images of immunocytochemistry of MYOD1 in DMD and CORR-R3381X MPCs and at 24, 48 and 120h in secondary differentiation medium. Scale bars are 50 µm.

(C) Representative images of immunocytochemistry of MYH stained with MF20 antibody in DMD and CORR-R3381X MPCs and at 24, 48 and 120h in secondary differentiation medium. Scale bars are 50 µm.

(D) Log_2RPKM values of *PAX7* gene do not significantly differ in DMD and CORR-R3381X muscle cells at MPCs (0h) stage, after 24 and 120h in secondary differentiation medium. N=3, values are mean ± SD. Two-way ANOVA followed by Sidak's multiple comparisons test.

(E) Log_2RPKM values of *MYOD1* gene follow a similar trend in DMD and CORR-R3381X muscle cells at MPCs (0h) stage, after 24 and 120h in secondary differentiation medium. N=3, values are mean ± SD. Two-way ANOVA followed by Sidak's multiple comparisons test.

(F) Log_2RPKM values are significantly lower in DMD-R3381X muscle cells at the three stages of secondary differentiation when compared with CORR-R3381X cells. N=3, values are mean ± SD. Two-way ANOVA followed by Sidak's multiple comparisons test, ****$p < 0.0001$.

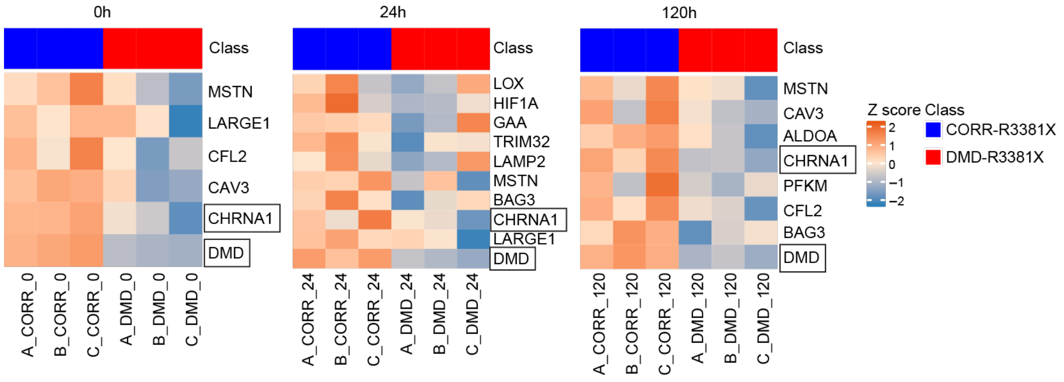

Fig. S3. Heatmaps of core enrichment genes in GO MUSCLE CELL CELLULAR HOMEOSTASIS at 0, 24 and 120 hours. Both *DMD* and *CHRNA1* are down-regulated in DMD-R3381X compared with CORR-R3381X.

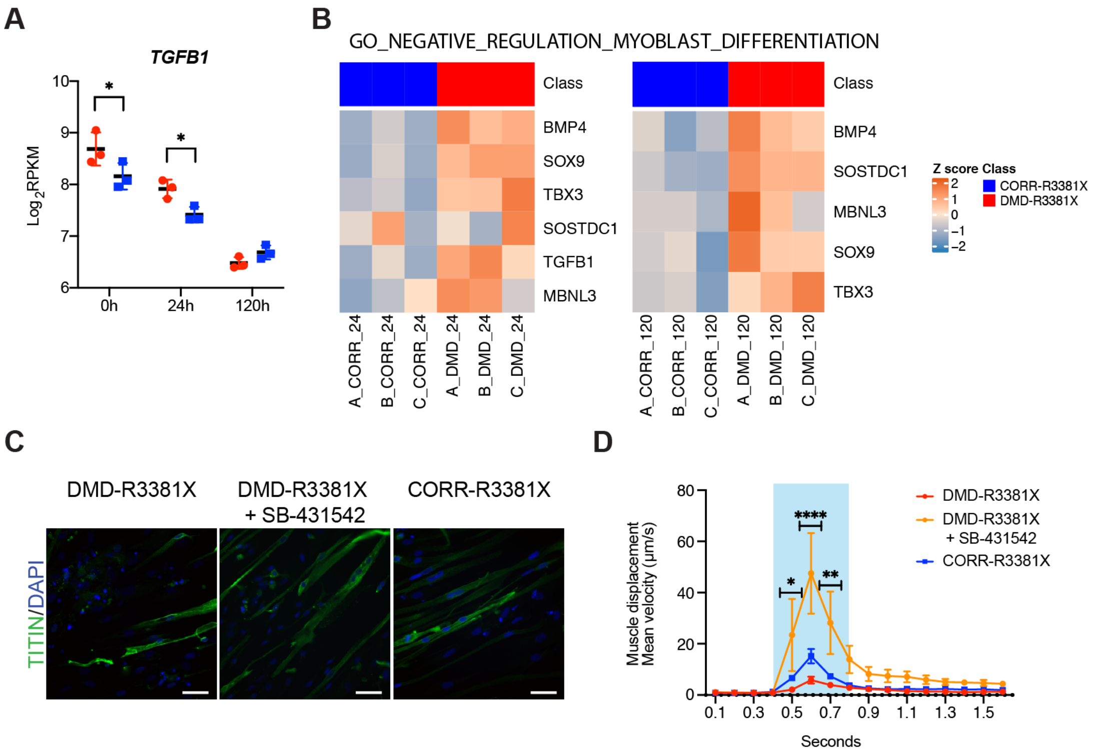

Fig. S4. SB-431542 treatment of DMD-R3381X muscle cells during secondary differentiation

(A) Log_2RPKM values of *TGFB1* are significantly higher in DMD-R3381X MPCs during secondary differentiation. N=3, values are mean ± SD. Two-way ANOVA followed by Sidak's multiple comparisons test, $*p < 0.05$.

(B) Heatmaps of core enrichment genes in GO NEGATIVE REGULATION OF MYOBLAST DIFFERENTIATION include *TGFB1* and genes involved in TGFβ signaling, which are up-regulated in DMD-R3381X at 24 and 120 hours of secondary differentiation.

(C) Representative images of immunocytochemistry for titin in 2D myogenic cultures of DMD-R3381X, DMD-R3381X treated with 10 µM SB-431542 and CORR-R3381X after 120h in secondary differentiation medium. Scale bars are 50 µm.

(D) Quantification of mean velocity of DMD-R3381X, DMD-R3381X + SB-431542 and CORR-R3381X myofibers upon optogenetic stimulation at day 5. The blue shading indicates the time during optogenetic stimulation. N=12. Values are mean ± SEM, Two-way ANOVA followed by Sidak's multiple comparisons test between DMD-R3381X + SB-431542 and CORR-R3381X samples. $*p < 0.05$, $**p < 0.01$, $****p < 0.0001$. The data is the same as in Figure 4D and 4F.

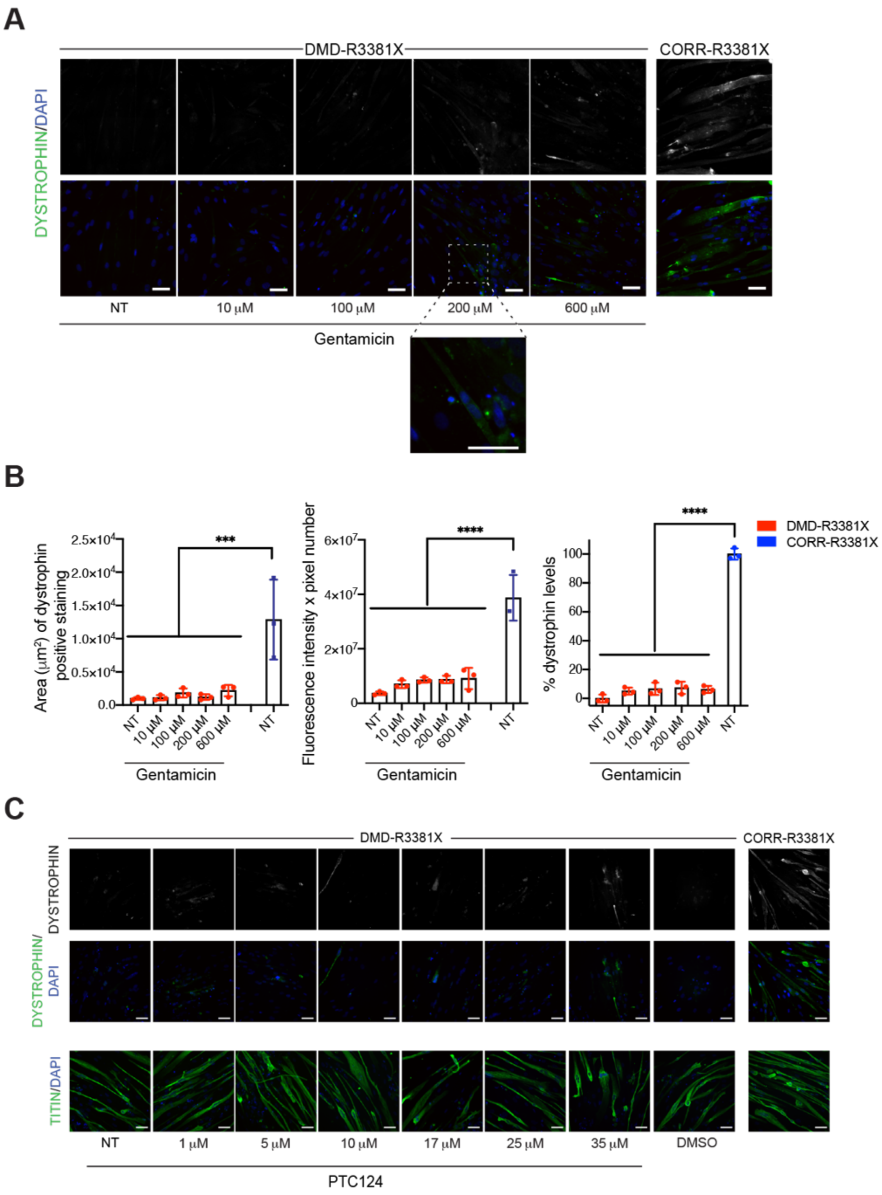

Fig. S5. Gentamicin or PTC124 treatment of DMD-R3381X muscle cells during secondary differentiation

(A) Representative immunocytochemistry images of dystrophin staining in DMD-R3381X, DMD-R3381X treated with a range of Gentamicin concentrations (10 µM, 100 µM, 200 µM and 600 µM) and CORR-R3381X cells after 120h in secondary differentiation medium. Scale bars are 50 µm.

(B) Quantification of percentage of dystrophin-positive area, mean fluorescence intensity multiplied by pixel number and percentage of normalized dystrophin levels in gentamicin treated

conditions. NT, Not treated. N=3. Values are mean ± SD. One-way ANOVA followed by Tukey's multiple comparisons test, ***$p < 0.001$, ****$p < 0.0001$.

(C) Representative immunocytochemistry images of dystrophin staining in DMD-R3381X, DMD-R3381X treated with a range of PTC124 concentrations (1 μM, 5 μM, 10 μM, 17 μM, 25 μM and 35 μM) and CORR-R3381X cells after 120h in secondary differentiation medium. Scale bars are 100 μm.

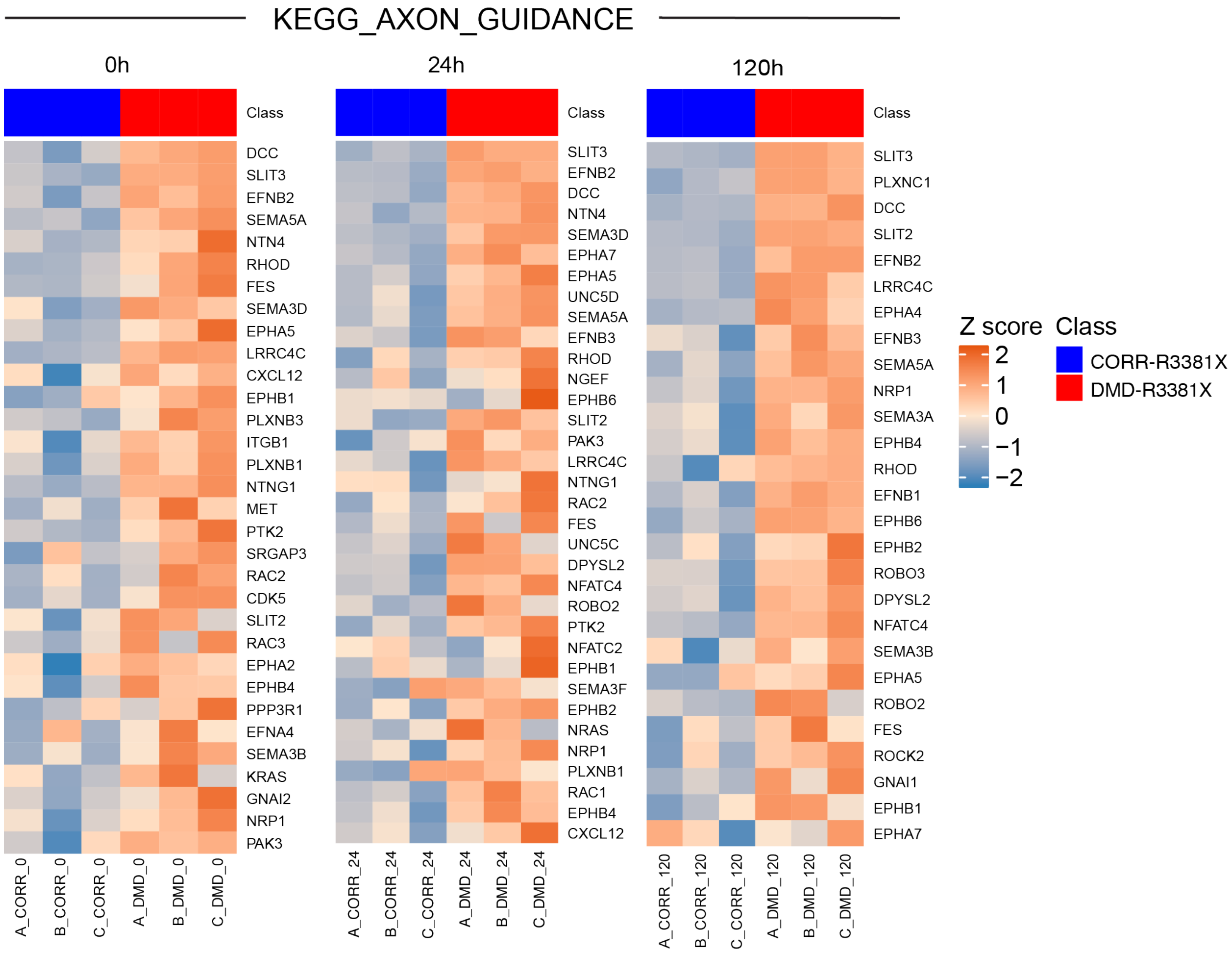

Fig. S6. Heatmaps illustrating log_2RPKM gene expression (row z-scores) of core enrichment genes for KEGG AXON GUIDANCE at each individual time point, columns represent samples and rows represent genes.

Table S1. Summary of DMD-R3381X patient's mutation, symptoms and the reprogrammed ePSC line

	P1
Fibroblasts	FB763 (P4)
Mutation	c.10141C>T (p.R3381X)
Exon	70
Ensembl variant ID	rs104894790
Sex	Male
Age at biopsy	6 years old
Muscular symptoms	Frequent falls
Microscopic description	Abnormal round fibre size, necrosis, increased internal nuclei, increase in fat/connective tissue,
Dystrophin immunocytochemistry	Absent
CK<br>Normal range<200 IU/L	10,000 IU/L
Brain symptoms	Severe learning difficulties
Cardiac symptoms	N/A
PSC type	ePSCs
PSC growth medium	EPSCM
PSC line	DMD-R3381X

Table S2. Primary antibodies

Antibodies	Species	Type	Isotype	Supplier	Cat Number	Working Dilution
OCT4	Mouse	Monoclonal	IgG2b	Santa Cruz	sc-5279	1:100
NANOG	Rabbit	Polyclonal	IgG	Abcam	AB80892	1:100
SOX2	Mouse	Monoclonal	IgG2a	R&D	MAB2018	1:100
TRA-1-60	Mouse	Monoclonal	IgM	Santa Cruz	sc-21705	1:100
SSEA4	Mouse	Monoclonal	IgG3	BD Bioscience	560796	1:50
α-Smooth Muscle Actin	Mouse	Monoclonal	IgG2a	R&D	MAB1420	1:75
β-III tubulin (TUBB3)	Mouse	Monoclonal	IgG2a	R&D	MAB1195	1:100
α-Fetoprotein	Mouse	Monoclonal	IgG1	R&D	MAB1368	1:100
PAX7	Mouse	Monoclonal	IgG1	DSHB	N/A	1:100
MYOD1	Mouse	Monoclonal	IgG1	Dako	M3512	1:100
MYH (MF20)	Mouse	Monoclonal	IgG2b	DSHB	N/A	1:100
Titin	Mouse	Monoclonal	IgM	DSHB	N/A	1:100
Dystrophin (Immunocytochemistry)	Mouse	Monoclonal	IgG2a	Millipore	MABT827	1:50
Dystrophin (Immunoblotting)	Rabbit	Polyclonal	IgG	Fisher Scientific	PA5-32388	1:750
β-Actin	Mouse	Monoclonal	IgG2a	Sigma	A5316	1:5,000
Vinculin	Mouse	Monoclonal	IgG1	Sigma	MAB3574	1:1000
Acetylcholine receptor, nicotinic, muscle	Rat	Monoclonal	IgG1	DSHB	mAb 35	1:200
Synaptic vesicle glycoprotein 2A	Mouse	Monoclonal	IgG1	DSHB	SV2	1:500

Table S3. Secondary antibodies

Antibodies	Species	Supplier	Cat Number	Working Dilution
Anti-mouse IgG1 488	Goat	Invitrogen	A-21121	1:1,000
Anti-mouse IgG 488	Goat	Invitrogen	A-28175	1:1,000
Anti-rabbit IgG 488	Goat	Invitrogen	A-11034	1:1,000
Anti-mouse IgG2a 488	Goat	Invitrogen	A-21131	1:1,000
Anti-mouse IgG2b 546	Goat	Invitrogen	A-21143	1:1,000
Anti-rat IgG 555	Goat	Invitrogen	A-21434	1:1,000
Anti-mouse IgM 594	Goat	Invitrogen	A-21044	1:1,000
Anti-mouse IgG1 647	Goat	Invitrogen	A-21240	1:1,000
IRDye 680RD Anti-mouse IgG	Goat	LI-COR Bioscience	926-68070	1:10,000
IRDye 800CW Anti- rabbit IgG	Donkey	LI-COR Bioscience	926-32213	1:10,000

Table S4. RT-qPCR primers list

Target Gene	Sequence (5'-3')
NANOG	F-AGAAAAACAACTGGCCGAAGAAT
	R-GTTGAATTGTTCCAGGTCTGGTT
OCT4	F-CACTGTACTCCTCGGTCCCTTTC
	R-CAACCAGTTGCCCCAAACTC
TGFB1	F- TCGCCAGAGTGGTTATCTT
	R- TAGTGAACCCGTTGATGTCC
MUSK	F- GCCTTCAGCGGAACTGAGAAA
	R- GGCTGGGGGTAGGATTCCA
SLIT2	F- GACGACTGCCAAGACAACAA
	R- TGATAGCCAGGCAAACACTG
SLIT3	F- AGCGCCTTGACCTGGACA
	R- TCGGCGTGCTCTGGAAAA
ROBO2	F- GGGTTACTACATCTGCCAGGCTT
	R- AGGTGGAGGTCTATCTGTCAAAACAT
EFNB2	F- GCAAGTTCTGCTGGATCAAC
	R- AGGATGTTGTTCCCCGAATG
EPHB4	F- GTCTGACTTTGGCCTTTCCC
	R- TGACATCACCTCCCACATCA
SEMA3D	F- TGGGACATCGAAGACAGCAT
	R- AAAGTGTGCTCCTGGGCTTT
SEMA5A	F- GTCTATACTTACTGCCAGCG
	R- GTTAAATGCCTTGATGGCCTC
ACTB	F- GCGAGAAGATGACCCAGATC
	R- CCAGTGGTACGGCCAGAGG

Table S5. Oligonucleotide primer sequences used to amplify the fragments for Gibson Assembly

Fragment	Sequence (5'-3')	Product Length (bp)
Left Arm	F-CGCGCCGGTACCTTAATTAAACTAAATGCTAGGCATTTAC	1,040
	R-GACTATCTTTCTAGGGTTAAGGAGAGTGTTGTGGTTGTGA	
Right Arm (1)	F-TGATCTCACCATGATCTCCCTTTTAGACTACATCAGGAGAAGATGTTCGAGACTTTGCCAAGGTACTAAAAAACAAATTTCGAACCAAAAGGTATTTTGC	950
	R-GGGGATCCACTAGTTCTAGAGCAGCACCCTTCAGCAAAAA	
Right Arm (2)	F-GATTATCTTTCTAGGGTTAATTACAAAACAAGTGTCATGGGGCAGAAGACTGGAGTGGTCATTAGTTTTGAAATCATCCTGTCCTAAATCTGATCTCACC	1,040
	R-GGGGATCCACTAGTTCTAGAGCAGCACCCTTCAGCAAAAA	
Backbone Vector	F-TTTTTGCTGAAGGGTGCTGCTCTAGAACTAGTGGATCCCC	3,013
	R-GTAAATGCCTAGCATTTAGTTTAATTAAGGTACCGGCGCG	
Selection Cassette	F-TCACAACCACAACACTCTCCTTAACCCTAGAAAGATAGTC	3,277
	R-CCATGACACTTGTTTTGTAATTAACCCTAGAAAGATAATC	

Right arm (1) primer was used to introduce the corrected base (green underlined).

Other Supplementary Materials for this manuscript include the following:

Movie S1. DMD-R3381X contraction video_S1 for Fig 3

Movie S2. CORR-R3381X contraction video_S2 for Fig 3

Movie S3. DMD-R3381X contraction video_S3 for Fig 4

Movie S4. DMD-R3381X + SB-431542 contraction video_S4 for Fig 4

Movie S5. CORR-R3381X contraction video_S5 for Fig 4

Movie S6. CORR-R3381X + SB-431542 contraction video_S6 for Fig 4

Data S1. gsea_report_Mut_vs_Ctrl_cp

Data S2. gsea_report_Mut_vs_Ctrl_gobp
